# Sepsis Induces Long‐Term Muscle and Mitochondrial Dysfunction due to Autophagy Disruption Amenable by Urolithin A

**DOI:** 10.1002/jcsm.70041

**Published:** 2025-08-15

**Authors:** Alexandre Pierre, Raphael Favory, Benoit Brassart, Claire Bourel, Raphael Romien, Sylvain Normandin, Arthur Dubech, Claire Vincent, Jeremy Lemaire, Gaelle Grolaux, Ophelie Not, Frederic Wallet, Frederic Daussin, Eric Boulanger, Arthur Durand, Marie Frimat, Alina Ghinet, Michael Howsam, William Laine, Philippe Marchetti, Jerome Kluza, Estelle Chatelain, Jimmy Vandel, Nicolas Barois, Valerie Montel, Bruno Bastide, Sebastien Preau, Steve Lancel

**Affiliations:** ^1^ Univ. Lille, Inserm, CHU Lille, Institut Pasteur de Lille, U1167‐RID‐AGE‐Facteurs de Risque et Déterminants Moléculaires des Maladies Liées au Vieillissement Lille France; ^2^ Division of Intensive Care, Hôpital Roger Salengro CHU de Lille Lille France; ^3^ Division of Bacteriology, Biology Pathology Institute of Lille CHU de Lille Lille France; ^4^ Univ. Lille, Univ. Artois, Univ. Littoral Côte D'Opale, ULR 7369‐URePSSS ‐ Unité de Recherche Pluridisciplinaire Sport Santé Société Lille France; ^5^ Division of Geriatrics, Hôpital Claude Huriez CHU de Lille Lille France; ^6^ Division of Nephrology, Hôpital Claude Huriez CHU de Lille Lille France; ^7^ Health and Environment, Laboratory of Sustainable Chemistry and Health Junia Lille France; ^8^ Faculty of Chemistry Alexandru Ioan Cuza’ University of Iasi Iasi Romania; ^9^ Univ. Lille, CNRS, Inserm, CHU Lille, UMR9020‐U1277‐CANTHER‐Cancer Heterogeneity Plasticity and Resistance to Therapies Lille France; ^10^ Tissue Bank, Biology Pathology Institute of Lille CHU Lille Lille France; ^11^ Univ. Lille, CNRS, Inserm, CHU Lille, Institut Pasteur de Lille Lille France; ^12^ Univ. Lille, CNRS, Inserm, CHU Lille, Institut Pasteur de Lille, U1019‐UMR 9017‐CIIL‐Center for Infection and Immunity of Lille Lille France

**Keywords:** autophagy, mitochondria, muscle, sepsis

## Abstract

**Background:**

Sepsis survivors often experience sustained muscle weakness, leading to physical disability, with no pharmacological treatments available. Despite these well‐documented long‐term clinical consequences, research exploring the cellular and molecular mechanisms is sorely lacking.

**Methods:**

Bioinformatic analysis was performed in the *vastus lateralis* transcriptome of human ICU survivors 7 days after ICU discharge (D7), 6 months (M6) and age‐ and sex‐matched controls. Enrichment analysis using Gene Ontology (GO) terms and Mitocarta3.0 was performed at D7 and M6 on differentially expressed genes (DEGs) and modules identified by weighted gene co‐expression network analysis (WGCNA). Using a murine model of resuscitated sepsis induced by caecal slurry injection, pathways identified by the bioinformatics analysis were explored in 18‐ to 24‐week‐old sepsis‐surviving (SS) mice at Day 10. Autophagy flux was investigated both in vivo and in vitro with chloroquine, a lysosomal inhibitor and urolithin A (UA), an autophagy inducer. Systemic metabolism was evaluated with indirect calorimetry, muscle phenotype with in situ and ex vivo contractility, muscle mass, myofibre cross‐sectional area and typing and mitochondrial population with transmission electron microscopy (TEM), as well as mitochondrial function with high‐resolution respirometry. Autophagic vacuole (AV) level was monitored using LC3B‐II and P62 protein expression and TEM.

**Results:**

Pathways related to ‘mitochondrion’ were the only ones whose deregulation persisted between D7 and M6 (*p* < 0.05) and characterized WGCNA modules correlated with muscle mass, strength and physical function. Shared mitochondrial DEGs between D7 and M6 encoded matrix mitochondrial proteins related to ‘metabolism’ and ‘mitochondrial dynamics’. SS mice exhibited reduced complex I‐driven oxygen consumption (CI‐J_O2_) (−45%), increased S‐nitrosylation of complex I, damaged (+35%) and oxidized (+51%) mitochondria and AV accumulation (5 vs. 50 AVs/mm^2^) compared with sham pair‐fed mice (*p* < 0.05) despite no differences in mitochondrial size or number. Autophagy flux was reduced in SS mice due to decreased AV degradation ratio (*p* < 0.05). UA restored a balanced autophagy flux (turnover ratio 0.96 vs. −0.17) by increasing AVs formation and degradation ratio (*p* < 0.05). UA also improved CI‐J_O2_ (81 vs. 106 pmol/s/mg), tetanic force (215 vs. 244 mN/mm^2^) and hindlimb muscle weight in SS mice (*p* < 0.05).

**Conclusion:**

Mitochondrial and autophagy disruption contributes to long‐term muscle dysfunction in human and mouse sepsis survivors. We demonstrate for the first time that sepsis induces an autophagy flux blockade. Urolithin A prevents mitochondrial and muscle impairments both in vivo and in vitro by improving autophagy flux.

## 1 Introduction

Survivors of sepsis—a severe infection which triggers an inappropriate, acute inflammatory response resulting in admission to an intensive care unit (ICU)—are increasingly numerous (39 million worldwide/year), in part because of improved diagnosis and clinical care [1, 2]. However, sepsis is responsible for several consequences after an ICU stay, known collectively as post‐intensive care syndrome, which impairs long‐term prognoses [3]. Unravelling the underlying mechanisms is a prerequisite to developing tools helping to recover from sepsis [4]. A predominant feature among survivors is the reduction of muscle strength during their hospital stay that persists for months after ICU discharge and results in reduced capacity for exercise, long‐lasting physical disability, distress and a higher risk of mortality up to 5 years after discharge [5, 6]. It also results in significant healthcare costs and is rapidly becoming a major public health challenge [3]. However, there is currently no pharmacological treatment, and research exploring the cellular and molecular mechanisms is sorely lacking.

Although mitochondrial dysfunction is a hallmark of sepsis‐induced muscle dysfunction in the acute phase [7], little is known about its persistence in the convalescence phase. Among others, Dos Santos et al. investigated this hypothesis in 2016 and found no difference using electron microscopy in the mitochondrial content of quadriceps tissue among ICU survivors compared with healthy volunteers [8]. Nevertheless, mitochondrial function can be compromised in some conditions, even without a reduction in mitochondrial quantity [9]. Owen et al. reported reduced oxidative phosphorylation (OXPHOS) capacities of isolated mitochondria in the skeletal muscles of murine sepsis survivors [10]. Recently, Mart et al. showed that human sepsis survivors exhibited cardiopulmonary physiological responses to exercise similar to those observed in non‐critically ill patients with mitochondrial myopathies [11]. But the impact of mitochondrial dysfunction on muscle function remains to be demonstrated, and a more comprehensive grasp of the relevant cellular and molecular processes could facilitate the development of a therapeutic approach to counteract post‐sepsis muscle weakness.

The present study investigates the causal relationship between mitochondrial and muscle dysfunction and elucidates the underlying mechanisms.

## 2 Methods

Detailed protocols, antibodies and reagents are provided in the Supporting Information.

### 2.1 Bioinformatic Analysis

Human quadriceps muscle gene expression dataset was sourced from GEO (GSE6011). Lists of differentially expressed genes (DEGs) for D7 versus CTRL and M6 versus CTRL and Gene Co‐expression Network Analysis (WGCNA) gene modules from the study by Walsh et al. [12] were analysed for enrichment using R (v4.3.1) with gprofiler (v0.2.3) to scan GO:BP, GO:CC, GO:MF, KEGG and HP pathways, and clusterProfiler (v4.10.0) to scan Human MitoCarta3.0 pathways [13]. Muscle and physical dysfunction were defined by Walsh et al. as a reduced muscle mass (measured by quadriceps' cross‐sectional area [CSA]), muscle strength (measured by MRC sum score or peak torque) and physical function (assessed by Functional Independence Measure) [12].

### 2.2 The Murine Sepsis Model With Delayed Resuscitation

Sepsis was induced via intraperitoneal (IP) caecal slurry injection (CSI) in 18‐ to 24‐week‐old male and female C57BL/6J mice, with sham mice receiving glycerol‐PBS. Mice were resuscitated with antibiotics and fluids from the 12‐h post‐CSI for 5 days to mimic human temporality. Surviving‐sepsis (SS) mice were analysed at Day 10. (1) To assess the impact of the energy intake reduction on muscle phenotype, mice were divided into three groups: sham fed (SF), sham pair‐fed (SPF) and sepsis. (2) To assess autophagy flux in skeletal muscle, mice were treated with chloroquine (CQ), a lysosome inhibitor [14], and divided into four groups: SPF vehicle (Ve), SPF CQ, Sepsis Ve and Sepsis CQ. (3) To assess whether increasing autophagic flux improved muscle phenotype, we used urolithin A (UA), a natural compound known to induce selective and non‐selective autophagy [15, 16]. Mice were divided into four groups: SPF placebo (Pl), SPF UA, sepsis Pl and sepsis UA. To evaluate whether UA increased autophagy flux in our model, UA and CQ were concomitantly administered in SPF and sepsis mice. Muscle phenotype was assessed with muscle mass, myofibre CSA and typing, in situ and ex vivo contractility, mitochondrial respiration, DNA copy number and oxidation. Autophagy vacuole (AV) level was monitored using transmission electron microscopy (TEM) and microtubule‐associated protein 1 light chain 3 B‐II (LC3B‐II) and P62 protein expression. Autophagy flux was analysed following the method of Plaza‐Zabala et al. [17]. All animal procedures were approved by the local ethics committee (Animal Experimentation Ethics CEEA75, Lille, France, APAFIS#18107‐2018121214148408).

### 2.3 Cell Culture

C2C12 myoblasts (ATCC CRL‐1772) were cultured in Dulbecco's Modified Eagle Medium high glucose supplemented with 10% fetal bovine serum and 1% penicillin–streptomycin, differentiated into myotubes over 7 days with 1% horse serum. Myotubes were treated with lipopolysaccharide (LPS), adenosine triphosphate (ATP), chloroquine and UA to assess autophagy flux and mitochondrial ROS production.

### 2.4 Statistical Analysis

All statistical analyses were performed with GraphPad Prism 10 (GraphPad, San Diego, USA). Kaplan–Meier survival curves were analysed using the log‐rank test, whereas group comparisons were conducted using parametric tests (Student *t*‐test, one‐way ANOVA and Welch's ANOVA) or non‐parametric tests (Mann–Whitney and Kruskal–Wallis) based on data distribution and variance. Time‐based group comparisons employed two‐way ANOVA with post hoc Tukey correction. Comparisons with a *p* value < 0.05 were considered statistically significant.

## 3 Results

### 3.1 Dysregulation of Mitochondria‐Related Pathways Persists Over Time

**FIGURE 1:**
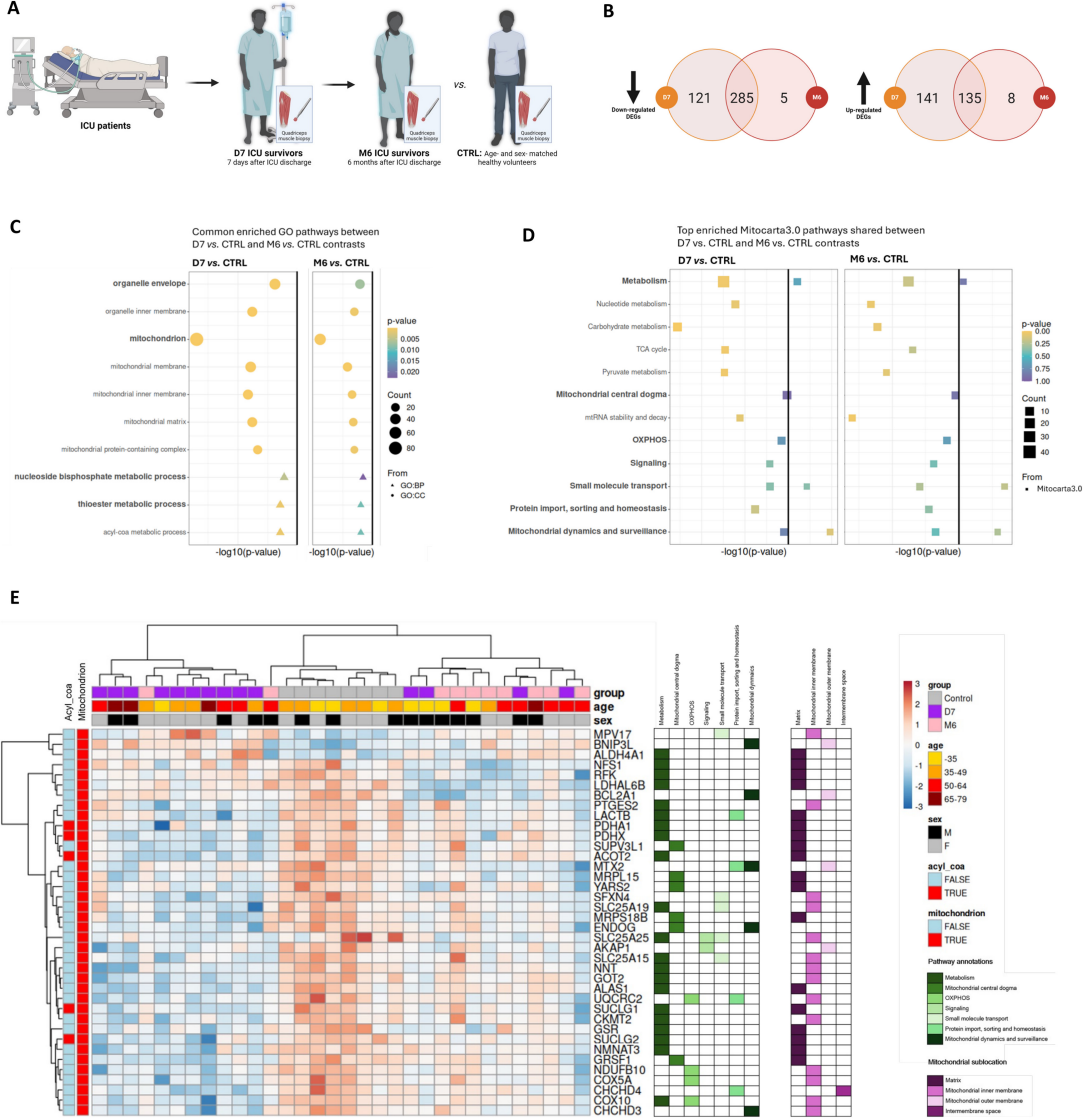
Sustained dysregulation of mitochondria‐related genes over time in human ICU survivors. (A) Schematic representation of the Dos Santos et al. study design: ICU survivors 7 days (D7, *n* = 14) and 6 months (M6, *n* = 10) after ICU discharge and age‐ and sex‐matched healthy volunteers (CTRL, *n* = 8) underwent muscle biopsy of the *vastus lateralis*. Created with BioRender. (B) Venn diagram representing the down‐regulated (left) and up‐regulated genes (right) in D7 versus CTRL and M6 versus CTRL contrasts. (C) Dot plot representing all the enriched GO pathways shared between down‐regulated genes for D7 versus CTRL (left) and M6 versus CTRL (right) contrasts. Dots indicate the GO category of the pathway and the gradient colour the adjusted *p* value of the enrichment. The principal pathways are in bold font and the sub‐pathways in non‐bold font. (D) Dot plot representing the enriched MitoCarta3.0 pathways shared between D7 and CTRL (left) and M6 and. CTRL (right) contrasts. All the principal mito‐pathways are shown in bold font. Only the top 5 sub mito‐pathways are shown in non‐bold font, representing the shared pathways between the two contrasts. Enrichments over down‐regulated genes are shown on the left side of the vertical solid line, whereas enrichments over up‐regulated genes are shown on the right side. (E) Gene expression heatmap of the common mitochondrial‐related DEGs in D7 versus CTRL and M6 versus CTRL contrasts, clustered over rows and columns according to gene expression. Additional annotations on genes (GO and MitoCarta3.0) and samples (age, sex and group) are displayed. Annotations of principal mito‐pathways and mitochondrial compartment localization according to Human MitoCarta3.0 are represented in green and purple colour gradients, respectively. BP, biological process; CC, cellular component; GO, Gene Ontology.

We conducted a comprehensive bioinformatic analysis of the publicly available muscle transcriptome data of ICU survivors suffering from sustained muscle weakness in the prospective cohort of Dos Santos et al. [8, 12]. Fourteen patients underwent *vastus lateralis* biopsy 7 days (D7) after ICU discharge and 10 of them again at 6 months (M6) and were compared with 8 age‐ and sex‐matched healthy volunteers (CTRL) (Figure 1A). Within this two‐stage, longitudinal study framework, we initially analysed the DEGs, then the gene modules discovered by WGCNA, that were associated with both muscle and physical dysfunction. We found that 285 down‐regulated and 135 up‐regulated genes were common when comparing D7 versus CTRL and M6 versus CTRL contrasts (Figure 1B). A first enrichment analysis with Gene Ontology (GO) terms revealed that the top 20 pathways were predominantly related to mitochondria or metabolism at D7 and M6 (Figure S1). The most significantly enriched pathways shared between D7 and M6 were ‘mitochondrion’ in the GO cellular component (CC) category and ‘acyl‐coA metabolic process’ in the GO biological process (BP) category (Figure 1C). To increase the specificity of these results, a second enrichment analysis was performed using mitochondrial‐related pathways (mito‐pathways) annotated in Human MitoCarta3.0 [18], which revealed that ‘carbohydrate metabolism’ was the leading mito‐pathway at D7 and remained key at M6 (Figure 1D). This mito‐pathway was mainly related to ‘pyruvate metabolism’ and ‘TCA cycle’. Among the 38 shared mitochondrial‐related genes between D7 and M6 contrasts (35 down‐regulated and 3 up‐regulated), 47.4% encoded mitochondrial proteins located in the matrix and 36.8% in the inner membrane (Figure 1E), whereas 55.3% were related to ‘metabolism’ and 15.8% to ‘mitochondrial dynamics and surveillance’ mito‐pathways (Figure 1E). In addition, we further analysed 17 modules identified through the WGCNA), of which only 3 were associated with both muscle strength, muscle mass and physical functioning at D7 [12]. The enriched pathways associated with these modules were mainly related to mitochondria or metabolism based on GO term annotations and are described in detail in Figure S1. The most significant mito‐pathways for each of the 3 modules (mod) were related to ‘metabolism’ in mod1, ‘mitochondrial dynamics and surveillance’ in mod2 and ‘mitochondrial ribosome’ in mod4, based on MitoCarta3.0 annotations (Figure S1). Overall, mitochondrial and metabolism pathways were among the top enriched pathways at D7 or M6. Mitochondrial pathways were the only ones whose deregulation persisted between D7 and M6. The down‐regulated genes mainly affected those encoding mitochondrial proteins localized in the matrix and involved in acyl‐coA and carbohydrate metabolism. Finally, pathways linked to mitochondrial metabolism characterized the gene co‐expression networks associated with both muscle and physical dysfunction.

### 3.2 Mitochondrial Dysfunction Involves Fatty Acid and Carbohydrate Oxidation

**FIGURE 2:**
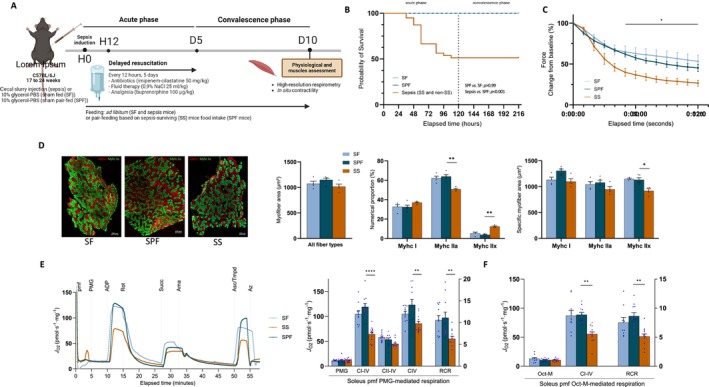
Mitochondrial dysfunction involves fatty acid and carbohydrate oxidation. (A) Design of the study: mice were subjected to intra‐peritoneal (i.p.) injection of 400 μL caecal slurry solution (100 mg/mL) (sepsis) or 10% PBS–glycerol solution (sham fed [SF] and sham pair‐fed [SPF]) at H0 and were resuscitated at H12. SF and Sepsis mice were fed *ad libitum*, and SPF mice received the same amount of food as the sepsis‐surviving (SS) mice. Muscles were harvested at D10. (B) Kaplan–Meier survival curve for SF mice (*n* = 18), SPF mice (*n* = 15), sepsis mice (*n* = 39). (C) In situ contractility: fatigability curve of *soleus* during 120 s stimulation at 40 Hz (*n* = 6–8 for each group). (D) Representative images of *soleus* myofibre types with Type I (red), Type IIa (green) or Type IIx (dark) myofibre. SF (left), SPF (middle), SS (right). Scale bars, 100 μm. *Soleus* global cross‐sectional area (CSA) (left), fibre type‐specific CSA (middle) and Myhc‐type proportions (right) (*n* = 4–5 for each group). (E) Representative respirometry profiles of *soleus* permeabilized muscle fibres (pmf) in SF mice (light blue curve), SPF mice (dark blue curve) and SS mice (orange curve). Oxygen consumption (J_O2_) recorded after sequential injections: pyruvate (5 mM), malate (2 mM) and glutamate (10 mM) (PMG); ADP (5 mM) (CI–IV for oxidative phosphorylation (OXPHOS) state driven by complex I); rotenone (Rot, 0.5 μM) and succinate (Suc, 10 mM) (CII–IV for OXPHOS state driven by complex II); antimycin A (Ama, 2.5 μM), ascorbate (Asc, 2 mM) and TMPD (0.5 mM) (CIV for OXPHOS state driven by CIV). PMG‐linked *soleus* pmf J_O2_ (*n* = 7–14 for each group). The respiratory control ratio (RCR) is plotted on the right Y‐axis in each graph. (F) *Soleus* pmf J_O2_ recorded after sequential injections: octanoyl‐carnitine (0.5 mM) and malate (2 mM) (Oct‐M) and then ADP (5 mM) (CI–IV) (*n* = 7–13 for each group). non‐SS, non‐surviving sepsis mice in dotted orange; SF, sham mice fed *ad libitum* in light blue; SPF, sham pair‐fed mice in dark blue; SS, sepsis‐surviving mice in orange. Each symbol represents one animal (panel E: male in blue and female in purple). Data expressed as means with SEM. Statistical comparison between SPF and SF (#) and SS and SPF (*), no comparison for the non‐SS group. Data analysed with log‐rank test (B), two‐way ANOVA test (C), one‐way ANOVA with post hoc Fisher's LSD test (E,F), Kruskal Wallis test with post hoc Dunn's test (D). *p < 0.05, **p < 0.01, ***p < 0.001, ****p < 0.0001.

To study metabolism and mitochondrial homeostasis in sepsis survivors, we used a murine model of resuscitated sepsis induced by CSI (Figure 2A) [19, 20]. Reduced calorie intake is a well‐known issue that may impact muscle function in human ICU survivors, as it is associated with low physical function scores at ICU discharge in a manner independent of illness severity [18]. To control the impact of this factor, we conducted pair‐feeding of sham (SPF) mice, restricting their diet to match that of sepsis‐surviving (SS) mice. We also analysed sham fed *ad libitum* (SF) mice as a control for SPF mice. The mortality rate was 51% in sepsis mice (Figure 2B), mimicking that of human septic shock [1]. Of key importance are the observations that no death occurred in the convalescence phase and clinical signs of infection were completely absent in SS mice (Figure S2), underlining the relevance of the CSI model in the assessment of sepsis survivorship. We also explored the muscle phenotype: In situ contractility experiments in anaesthetized mice revealed that the *soleus* force among SPF mice was not different from SF mice, but that the 100 Hz tetanic and 40 Hz endurance forces of SS mice were lower by 55% and 40%, respectively, compared with SPF mice (Figures 2C and S3). Ex vivo contractility of *extensor digitorum longus* (EDL) was not impaired in SS mice (Figure S3), indicating that muscle fibre type influenced the extent of muscle dysfunction. Muscle wasting was observed among SS mice's *tibialis anterior*, *gastrocnemius* and quadriceps muscles but not their *soleus* or EDL (Figure S3). These results demonstrate that the CSI model reproduced the sepsis‐induced muscle dysfunction observed in humans [5, 8], which was not wholly explained by the calorie intake reduction in SPF mice. In addition, the global CSA of *soleus* was preserved in SS mice (Figure 2D), indicating that the sepsis‐induced muscle dysfunction was not due to myofibre atrophy. *Soleus* myosin staining revealed a lower proportion of Type IIa fibres and a higher proportion of Type IIx fibres in SS mice compared with SPF mice, along with a reduced CSA of Type IIx fibres (Figure 2D). Similar reductions in Type IIb fibre CSA were also observed in the *tibialis anterior* (Figure S3). This shows that sepsis induced a shift in myofibre composition towards a more glycolytic phenotype in survivors.

Indirect calorimetry revealed that an energy deficit occurred in both SPF and SS mice (Figure S4). This deficit peaked earlier in the SPF group (Day 2) than in the SS group (Day 5). Nevertheless, body weight, lean mass and energy balance of both groups were restored in the convalescence phase (Figures S2 and S4). V_O2_ was higher and respiratory exchange ratio (RER) was lower in the SS group than in the SPF group around the 120th hour, the beginning of lean mass recovery (Figure S4), suggesting that sepsis modified the substrate oxidation response in sepsis survivors. To further explore the muscle oxidative capacities, we measured the mitochondrial respiration on permeabilized muscle fibres (pmf) of the *soleus* and EDL, as well as isolated mitochondria of the *tibialis anterior*. We found no difference in oxygen consumption (J_O2_) between SF and SPF mice. However, SS mice exhibited a reduction in J_O2_ compared with SPF mice, especially CI‐driven J_O2_ (Figure 2E,F). This reduction was observed regardless of the muscle type (*soleus*, EDL or *tibialis anterior*), substrate (PMG or Oct‐M) or method used (isolated mitochondria or pmf) (Figure S3). Overall, mitochondrial dysfunction, not attributable to reduced calorie intake, involved mainly complex I through both the fatty acid and carbohydrate oxidation pathways.

### 3.3 Damaged Mitochondria Accumulate Without Reduction in Biomass

**FIGURE 3:**
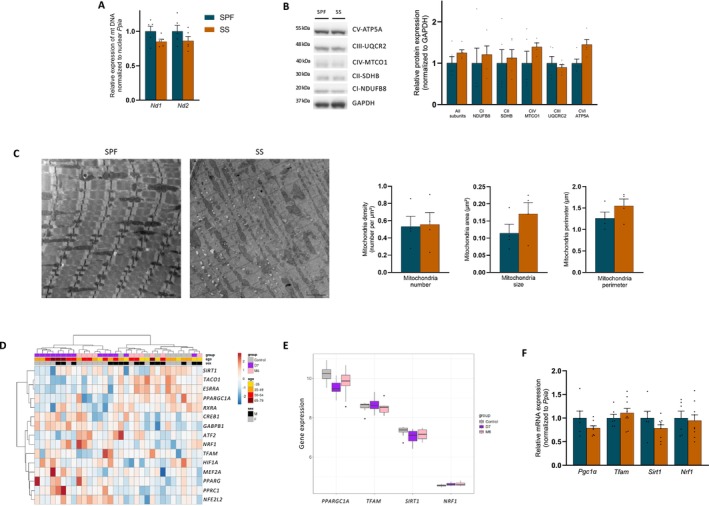
Mitochondrial biomass is retained. (A) Mitochondrial DNA (mt DNA) content measured by relative *Nd1* and *Nd2* mt DNA expression normalized to nuclear *Ppia* in quadriceps (*n* = 5 for each group). (B) Protein expression of respiratory chain subunits (*n* = 5 for each group). (C) Representative low‐magnification electron micrographs (left). Scale bars: 2000 nm. Quantification of mitochondrial mass by TEM: mitochondria number, area, perimeter in *tibialis anterior* (*n* = 4 for each group) (right). (D) Heatmap of gene expression illustrating hierarchical clustering of 15 regulators of mitochondrial biogenesis in quadriceps of ICU survivors 7 days (D7, *n* = 14), 6 months (M6, *n* = 10) after ICU discharge and healthy volunteers (control, *n* = 8). Experimental groups are colour‐coded above the heatmap: control in grey, D7 in purple and M6 in pink. Rows (genes) and columns (samples) are clustered according to expression values, represented by a colour gradient. Additional annotations on samples (age and sex) are displayed. (E) Boxplots representing gene expression of four master regulators (*PPARGC1A*, *TFAM*, *SIRT1* and *NRF1*) of the biogenesis transcriptional program in D7 (purple) and M6 (pink) versus control (grey). Boxes indicate medians and interquartile ranges. (F) mRNA changes of four master regulators of the biogenesis transcriptional program for SPF mice (*n* = 5) and SS mice (*n* = 9). Murine study: SPF, sham pair‐fed mice in dark blue; SS, sepsis‐surviving mice in orange. Each symbol represents one animal, and data are expressed as mean values with SEM. Data analysed by Mann–Whitney test. *p < 0.05, **p < 0.01.

Mitochondrial dysfunction can result from altered mitochondrial renewal processes, including reduced mitochondrial biomass owing to biogenesis failure or impairment of mitochondrial removal by selective and non‐selective (i.e., bulk) autophagy dysfunction [21, 22]. Firstly, no significant differences were observed between SPF mice and SS mice in the mitochondrial DNA copy number (Figure 3A) or protein expression of selected subunits of the respiratory chain complexes in quadriceps, *Tibialis anterior* and EDL (Figures 3B and S3). TEM analysis did not reveal any differences in mitochondrial number, size or perimeter in the *tibialis anterior* (Figure 3C). In addition, the transcriptional program of mitochondrial biogenesis did not appear to be deregulated, as its main mRNA components were not differentially expressed between survivors and controls in either human (Figure 3D,E) or mouse (Figure 3F) quadriceps. In conclusion, muscle mitochondrial biomass and biogenesis signalling of sepsis survivors did not differ from controls.

**FIGURE 4:**
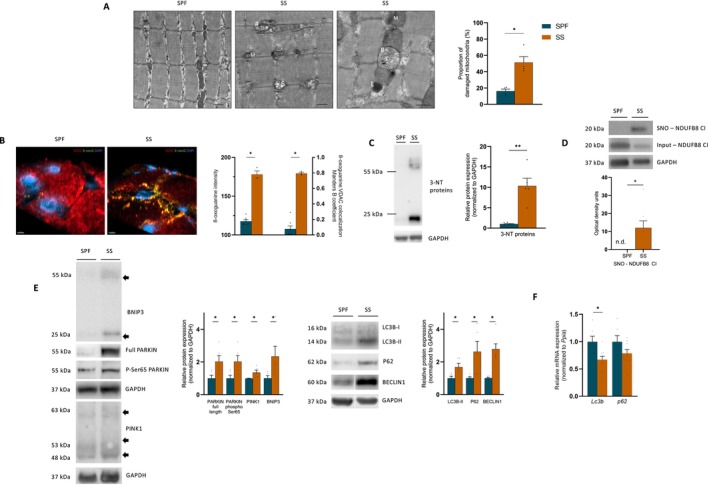
Mitochondria are damaged and oxidized. A, Representative micrographs illustrating intact mitochondria in SPF mice (M) (left), accumulation of damaged (#) and empty mitochondrial ($) (middle and right), lysosome (L) in *tibialis anterior*. Scale bars: 500 nm. Quantification of mitochondrial damage (*n* = 4 for each group). (B) Mt. DNA oxidation in *soleus*. Representative 3D images of nuclei, mitochondria and oxidized DNA staining respectively by DAPI (blue), VDAC (red) and 8‐oxoG (green) from SPF mice (*n* = 6, left), and SS mice (*n* = 3, right). 8‐oxoG intensity is plotted on the left Y‐axis and Manders' B coefficient of co‐localization on the right. (C) Expression of 3‐nitrotyrosine (3‐NT) proteins (*n* = 5 for each group). (D) Expression of S‐nitrosylated (SNO) NDUFB8 subunit complex I obtain after biotin switch assay and pull down for SPF mice (*n* = 5) and SS mice (*n* = 8). (E) Protein expression of PARKIN full length, PARKIN phospho Ser67, PINK1, BNIP3 (left) and LC3B‐II, P62, PARKIN, PINK1, BECLIN‐1 (right) in quadriceps (*n* = 5 for each group). (F) mRNA changes of *Lc3b* and *p62* for SPF mice (*n* = 5) and SS mice (*n* = 8). SPF, sham pair‐fed mice in dark blue; SS, sepsis‐surviving mice in orange. Each symbol represents one animal. Data expressed as mean values with SEM. Data analysed by Mann–Whitney test. *p < 0.05, **p < 0.01.

However, we still observed accumulation of damaged and oxidized mitochondria at Day 10 in septic mouse survivors, with a significantly higher proportion of aberrant mitochondria observed by TEM (Figure 4A), and increased 8‐oxoguanine co‐localized with mitochondria (Figure 4B) in the SS group than in the SPF group. Importantly, SS mice exhibited muscle nitrosative stress (Figure 4C), especially increased S‐nitrosylation of complex I (Figure 4D), an irreversible post‐translational modification known to alter complex I activity [23]. Such abnormalities may explain the complex I dysfunction and necessitate activation of autophagy to maintain mitochondrial homeostasis [21, 22]. The kinase phosphatase and tensin homologue‐induced kinase 1 (PINK1) and the E3 ubiquitin ligase PARKIN work together to detect the functional condition of mitochondria and tag impaired mitochondria for removal through autophagy, whereas BCL2/adenovirus E1B 19 kDa protein‐interacting protein 3 (BNIP3), a mitochondrial outer membrane protein, acts as a driver of receptor‐mediated mitophagy [21, 22]. Sepsis increased the protein expression of PINK1, Ser65 phospho PARKIN, full‐length PARKIN and BNIP3 in survivors (Figure 4E), suggesting that mitophagy signalling pathways were activated in response to mitochondrial damage. Similar results were found for BECLIN1, LC3B‐II and P62 proteins (Figure 4E), markers of the autophagic vacuoles. Whereas LC3B‐II and P62 protein levels increased, their mRNA expressions were not up‐regulated (Figure 4F), indicating that the elevation in protein levels may be attributed to an accumulation rather than increased synthesis. Taken together, these data suggest that mitochondrial dysfunction may not be related to reduced biomass per se, but rather to insufficient mitochondrial removal by autophagy.

### 3.4 Autophagy Flux Is Disrupted due to Reduced Degradation of Autophagic Vacuoles

**FIGURE 5:**
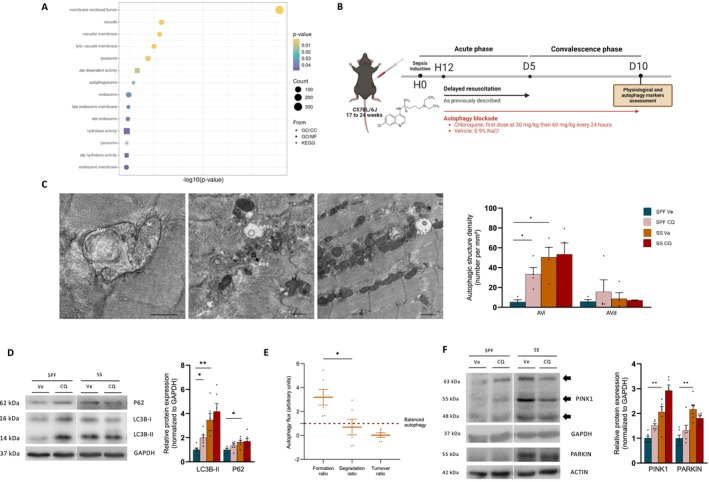
Autophagy flux is disrupted due to reduced degradation of autophagic vacuoles. (A) Dot plot representing the enriched autophagy‐related pathways in module 2 of WGCNA using GO:BP, GO:CC, GO:MF, KEGG and HP sets. (B) Design of the study: Mice were resuscitated and randomly assigned to Ve (0.9% NaCl) or CQ i.p. injection (first dose at 30 mg/kg and then 60 mg/kg/day) at the 12th hour for 10 days. (C) Representative micrographs of SS mice illustrating an autophagic vacuole engulfing a damaged mitochondria (left), numerous aberrant autophagic vacuoles (AVs) and autophagic debris (middle) in *tibialis anterior*. Black arrow indicates degradative autophagic vacuoles (AVd). Scale bars, 500 nm. Quantification of initial autophagic vacuoles (AVi) and AVd number (*n* = 4 for each group). (D) Protein expression of LC3B‐II and P62 in *soleus* (*n* = 6 for each group). (E) Calculation of the autophagy flux based on LC3B‐II expression. The turnover ratio represents the ratio between the formation ratio and the degradation ratio. The red dotted line represents a balanced autophagy turnover. (F) Protein expression of PINK1 and PARKIN in *soleus* (*n* = 6 for each group). CQ, chloroquine injection; SPF, sham pair‐fed mice; SS, sepsis‐surviving mice; Ve, vehicle injection. SPF Ve in dark blue, SPF CQ in light red, SS Ve in orange and SS CQ in dark red. Each symbol represents one animal. Data expressed as mean values with SEM. Data analysed by Kruskal–Wallis test with post hoc Dunn's test (C,D,F), Mann–Whitney test (E). *p < 0.05, **p < 0.01.

**FIGURE 6:**
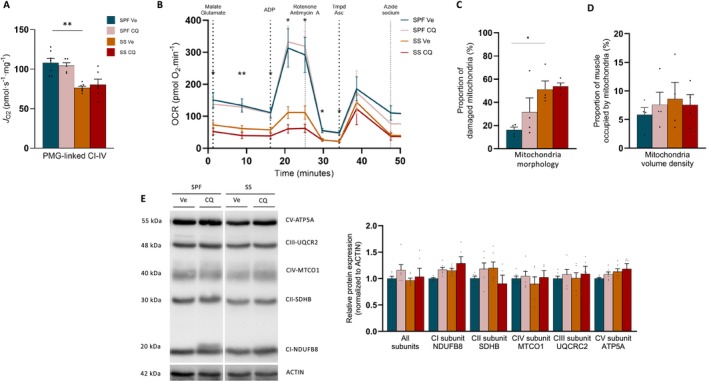
Pharmacological blockade of autophagy flux does not contribute to further mitochondrial damage. (A) PMG‐linked CI‐driven J_O2_ of *soleus* pmf (*n* = 6–7 for each group). (B) OCR curves of isolated mitochondria in *tibialis anterior* for SPF mice (*n* = 6–8 for each group). Stars indicate significant difference between OCR curves of SPF CQ mice and SS Ve mice. No difference for SPF Ve versus SPF CQ and SS Ve versus SS CQ. (C,D) Quantification of mitochondria damage (I) and content (J) by TEM analysis (*n* = 4 for each group). (E) Protein expression of five respiratory chain subunits (*n* = 6 for each group). CQ, chloroquine injection; SPF, sham pair‐fed mice; SS, sepsis‐surviving mice; Ve, vehicle injection. SPF Ve in dark blue, SPF CQ in light red, SS Ve in orange, SS CQ in dark red. Each symbol represents one animal. Data expressed as mean values with SEM. Data analysed by Kruskal–Wallis test with post hoc Dunn's test (A,C–E), Mann–Whitney test (F), two‐way ANOVA test with post hoc Tukey test (B). *p < 0.05, **p < 0.01.

The autophagy pathways, including ‘autophagosomes’, ‘lysosome’ and ‘hydrolase activity’, were among the enriched pathways in mod2 in the muscle of human ICU survivors and associated with muscle and physical dysfunction (Figure 5A). To explore the autophagy process, we investigated the autophagy flux in SS mice by administering CQ, the most commonly used autophagy inhibitor in animal models [24], although it has never, to our knowledge, been studied in sepsis models. Based on our preliminary experiments (Figure S5), we administered daily i.p. injections of CQ at a first dose of 30 mg/kg at the 12th hour after sepsis induction and then 60 mg/kg every 24 h for 10 days (Figures 5B and S5). CQ administration in SPF mice increased the number of initial autophagic vacuoles in the *tibialis anterior* (Figure 5C), as well as LC3B‐II protein expression in the *soleus* (Figure 5D) and quadriceps (Figure S5). It clearly demonstrated the effectiveness of this protocol for blocking autophagy flux because autophagosomes accumulated in the skeletal muscle of mice in receipt of CQ. The number of autophagic vacuoles (Figure 5C) and the protein expression of P62 and LC3B‐II (Figure 5D) in SS mice treated only with a vehicle (Ve) were not different from those of SPF CQ mice or SS CQ mice, indicating that autophagy was blocked at the terminal stages in SS Ve mice. The impairment of autophagy flux resulted from a decreased rate of autophagic vacuoles degradation relative to formation, leading to a low turnover (Figure 5E). The expression of PINK1 and PARKIN proteins also increased in SS mice compared with SPF Ve mice (Figure 5F). The blockade of autophagy by CQ in SS mice did not result in further mitochondrial damage or a deterioration in the function or biomass, as indicated by high‐resolution respirometry (Figure 6A,B), TEM (Figure 6C,D) and protein expression experiments (Figure 6E). These data raise the possibility that autophagy may be already completely blocked in SS mice, and overall, these findings indicate that the autophagy flux was disrupted due to a low degradative rate of autophagic vacuoles.

### 3.5 Promoting Autophagy Flux With Urolithin A Prevents Muscle Dysfunction

**FIGURE 7:**
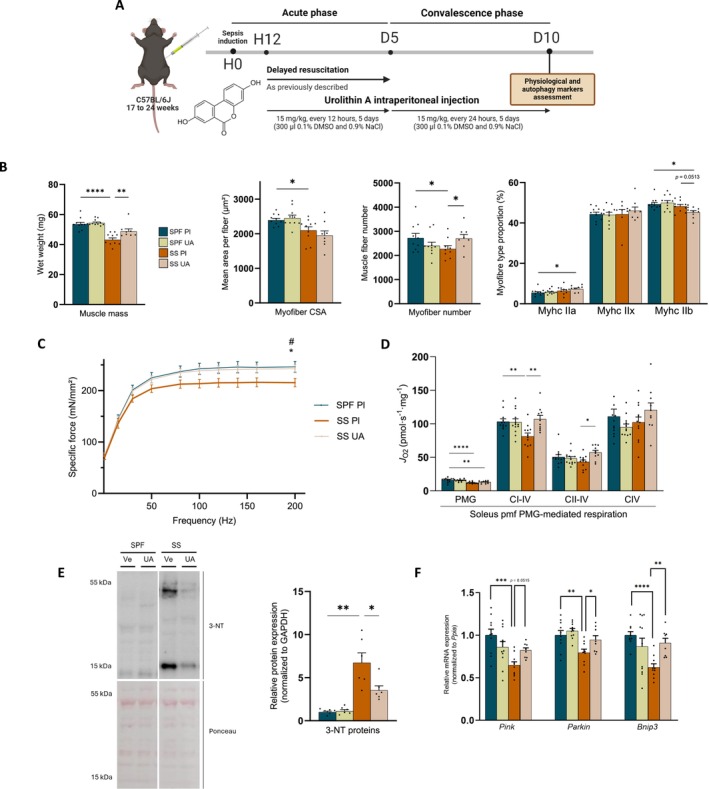
Urolithin A prevents muscle and mitochondrial impairments. (A) Design of the study: Mice were resuscitated and randomly assigned to Pl (300 μL of 0.1% DMSO and 0.9% NaCl) or UA i.p. injection (15 mg/kg every 12 h for 5 days and then every 24 h until day 10). (B) *Tibialis anterior* phenotype: muscle wet weight (left), myofibre CSA (middle left), myofibre number (middle right), myofibre type (right) (*n* = 8–10 for each group). One SS Pl value excluded due to cryosection‐related damage; exclusion did not affect the overall results. (C) Ex vivo contractility of soleus: specific force frequency curve measured at 1, 15, 30, 50, 80, 100, 120, 140, 160 and 200 Hz (*n* = 4–8 for each group). (D) PMG‐linked J_O2_ of *Soleus* pmf (*n* = 10–12 for each group). (E) Protein expression of 3 nitro‐tyrosine (*n* = 6 for each group). (F) mRNA changes of *Pink*, *Parkin* and *Bnip3* (*n* = 8–10 for each group). CQ, chloroquine; Pl, placebo; SPF, sham pair‐fed mice; SS, sepsis‐surviving mice; UA, urolithin A; Ve, vehicle. SPF Pl in dark blue, SPF UA in light yellow, SS Pl in orange and SS UA in light orange. Each symbol represents one animal. Data expressed as mean values with SEM. Data analysed by one‐way ANOVA test with post hoc Fisher's LSD test or Kruskal–Wallis test with post hoc Dunn's test (B,E), mixed‐effects model with post hoc Benjamini–Hochberg correction (F), Welch ANOVA test (F). # SPF versus SS Pl, *p < 0.05, **p < 0.01, ***p < 0.001, ****p < 0.0001.

To demonstrate that autophagy flux contributes to the pathogenesis of muscle and mitochondrial dysfunction in SS mice, we administered UA, obtained by eco‐catalysed Hurtley reaction (Figures 7A and S6) in the CSI‐induced sepsis mice. Although UA did not enhance clinical parameters or survival rate (Figure S7), it prevented the negative consequences of sepsis on muscle tissue. *Tibialis anterior* muscle dysfunction was characterized by reduced muscle mass and myofibre atrophy, as muscle wet weight, myofibre CSA and number in SS mice treated with Pl were reduced compared with SPF Pl (Figures 7B and S7). UA improved *tibialis anterior* muscle mass and myofibre number in SS mice, but not myofibre CSA (Figure 7B). Interestingly, UA induced a shift in myofibre towards a more oxidative phenotype among survivors, with higher IIa and lower IIx fibre proportions in SS UA mice than in SPF Ve mice (Figure 7B). Importantly, UA also prevented muscle and mitochondrial dysfunction in SS mice, as the tetanic force (Figures 7C and S7) and the PMG‐linked and OCT‐M‐linked CI‐driven respiration of *soleus* pmf were higher in SS UA mice than in SS Pl mice and were not different from SPF Pl mice (Figures 7D and S7). UA also reduced nitrosative stress in quadriceps of SS mice (Figure 7E), but these beneficial effects were not the result of an increase in mitochondrial biomass (Figure S7). Finally, UA influenced the mitophagy signalling pathways in SS mice, as it prevented the decrease in *Pink*, *Parkin* and *Bnip* mRNA changes observed in quadriceps of SS Pl mice (Figure 7F).

**FIGURE 8:**
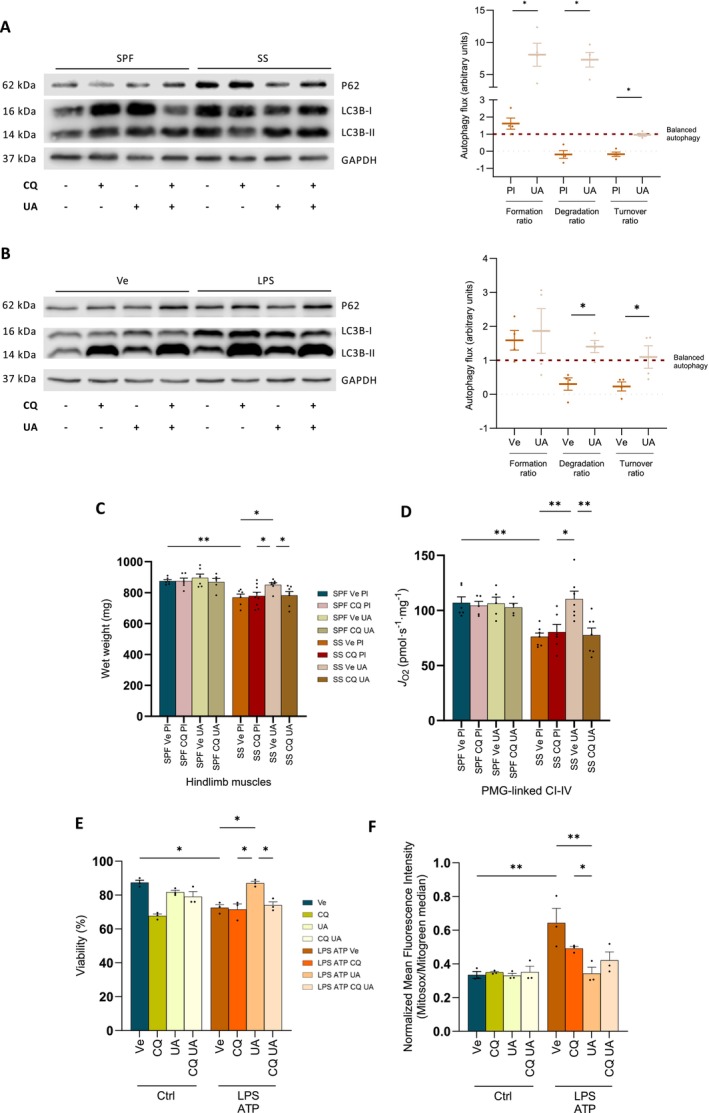
Urolithin A reduces muscle alterations by increasing autophagy flux. (A) Representative blot of LC3B‐II and P62 according to UA treatment with or without autophagy blockade by CQ in SPF and SS mice (*n* = 4 for each group). Calculation of the autophagy flux based on LC3B‐II expression. (B) Representative blot of LC3B‐II and P62 according to UA (50 μM) and CQ (50 μM) incubation in differentiated C2C12 cells challenged by LPS (500 ng/mL) (*n* = 4 for each group). Calculation of the autophagy flux based on P62 expression. (C) Wet weight of hindlimb muscles (*n* = 5–9 for each group). (D) PMG‐linked CI‐driven J_O2_ of *soleus* pmf (*n* = 5–7 for each group). (E,F) Cell viability (E) and Mitosox mean fluorescence intensity normalized to Mitogreen (F) in differentiated C2C12 cells challenged by LPS and ATP (*n* = 3 for each group). CQ, chloroquine; Pl, placebo; SPF, sham pair‐fed mice; SS, sepsis‐surviving mice; Ve, vehicle; UA, urolithin A. SPF Pl in dark blue, SPF UA in light yellow, SPF CQ in light red, SPF CQ UA in dark yellow, SS Pl in orange, SS UA in light orange and SS CQ UA in dark brown. Each symbol represents one animal or biological replicate. Data expressed as mean values with SEM. Data analysed by one‐way ANOVA test with Mann–Whitney test (A,B) and Kruskal–Wallis test with post hoc Dunn's test (C–F). *p < 0.05, **p < 0.01.

As UA improved the muscle and mitochondrial health in SS mice, we investigated whether its beneficial effects were mediated by autophagy. Using autophagy flux experiments [14, 17], we demonstrated that UA increased both the degradative and formation rates of autophagic vacuoles in the quadriceps of SS mice (Figures 8A and S8). The autophagic turnover rate of SS mice treated with UA, nearing a value of 1, was higher than that of SS mice treated with placebo. This indicates that UA restored a balanced autophagic flux in SS mice. In C2C12 muscle cells stimulated with LPS, one of the principal damage‐associated molecular pattern (DAMP) molecules implicated in sepsis pathophysiology, we confirmed that UA improved the degradative and turnover rates of P62, but not the rate of formation (Figures 8B and S8). Ultimately, although UA enhanced the hindlimb muscle wet weight and the PMG‐ and OCT‐M‐linked CI‐driven respiration in SS mice, these benefits were absent when autophagy flux was blocked by CQ (Figures 8C,D and S8). Consistent with these observations, UA increased cell viability and decreased the production of mitochondrial reactive oxygen species (ROS) in C2C12 cells challenged by LPS and ATP, an effect that was reduced with the blockade of autophagy flux by CQ (Figure 8E,F). These results demonstrate that the prevention of muscle and mitochondrial dysfunction by UA was indeed dependent on muscle autophagy. Overall, these experiments indicate that promoting autophagy flux with UA attenuated the sepsis‐induced muscle and mitochondrial dysfunction in murine survivors.

## 4 Discussion

We here provide novel evidence of the cellular and molecular basis of long‐term, sepsis‐induced muscle weakness so common among survivors of the condition. From among the hitherto poorly defined, multifaceted nature of this condition, encompassing aberrant muscle repair and impaired regeneration, mitochondrial dysfunction here emerges as a significant factor. Building upon the studies of Dos Santos et al. [8, 12], we have for the first time conclusively identified pathways that have long‐term impacts upon muscle homeostasis in patients who have suffered critical illness. In this two‐stage, longitudinal study, DEG analysis has revealed that mitochondrial‐related pathways were the only enriched pathways at both 7 days and 6 months after ICU discharge and that deregulation of these biochemical pathways is involved in the frequently observed persistence of post‐sepsis muscle dysfunction. To further elucidate these pathways we used the Human Mitocarta3.0 database, which provides an inventory of genes encoding proteins with inalienable sub‐mitochondrial compartment localization and pathways [13]. It led us to the discovery that the affected mitochondrial pathways were mainly related to acyl‐CoA metabolism, carbohydrate metabolism, pyruvate metabolism and the TCA cycle. Enriched metabolism mito‐pathways also characterized one module associated with both reduced muscle strength, muscle mass and physical functioning at D7 in the WGCNA analyses. Although mitochondrial pathways remained dysregulated, Dos Santos et al. reported that the mitochondrial content of the same patients was retained 6 months after ICU discharge [8]. Such a situation has also been reported in the skeletal muscle of septic patients with multiple organ failure [9]. A limitation of this study is the absence of clearly defined septic and non‐septic critically ill groups, making it difficult to discern whether the observed alterations are sepsis‐specific or related to critical illness. Future studies should include both to clarify sepsis‐specific mechanisms. This uncertainty, along with previous reports of mitochondrial functional impairment without a reduction in mitochondrial biomass [25], prompted us to further explore mitochondrial function in a murine model of resuscitated sepsis.

The CSI model was appropriate for the study of sepsis survivorship as SS mice showed no evidence of persistent peritoneal abscesses, demonstrating that the intra‐abdominal infection was successfully treated. This model also more than fulfilled the most recent ARRIVE guidelines and MQTiPSS recommendations for translational research in sepsis [26, 27], including delayed resuscitation, randomization and blinding. Both sexes were included, and findings in female mice were consistent with those observed in males, supporting the generalizability of our results. In addition, we have demonstrated here that the CSI model faithfully reproduced the human features of long‐term, sepsis‐induced muscle weakness [5]. Sepsis survivors exhibited impaired muscle contractility, regardless of muscle fibre trophicity, as evidenced by reduced force production in the soleus, despite preserved global CSA in the soleus. The atrophic response to sepsis was muscle type dependent and predominantly affected glycolytic fibres, as their fibre type‐specific CSA was reduced in both the soleus and *tibialis anterior*. Additionally, the soleus showed a glycolytic shift, a pattern recently reported in the quadriceps of ICU survivors [28]. In the cohort of Dos Santos et al., muscle mass did not correlate with muscle force and, consistent with our results, although some patients regained their muscle mass at 6 months post‐ICU, muscle weakness nevertheless persisted in ICU survivors [8]. These findings highlight an intrinsic defect in contractile properties of muscle fibres following sepsis, which may be linked to metabolic disorders. Using high‐resolution respirometry, we found that sepsis survivors exhibited mitochondrial dysfunction, which affected the functioning of the entire electron transfer chain, whatever the electron donor substrates. It affected both fatty acid and carbohydrate oxidation pathways and glycolytic, oxidative and mixed muscle types. Of primary importance is our finding that mitochondrial dysfunction was present without any reduction in mitochondrial biomass. This suggests that the quality of the mitochondrial population is at least as important, if not more so, than its quantity. Taken together, these data support the hypothesis of persistent, post‐sepsis mitochondrial dysfunction in both human and murine survivors.

As mitochondrial biomass and biogenesis were not different between SS and SPF mice, we focused on the other aspect of mitochondrial renewal, that is, mitochondrial removal, because its impairment also leads to mitochondrial dysfunction. When mitochondria are damaged, both selective and non‐selective (i.e., bulk) autophagy occur to restore a healthy and functional population [21]. Autophagy can be beneficial or deleterious to muscle health, depending on the disease and its stage of development [21], and there are also conflicting results within the field of sepsis [29]. Muscle autophagy signalling is mainly activated through Akt/mTOR inhibition and AMPK activation in the acute phase of sepsis, and it participates directly in muscle catabolism [29]. However, this does not mean that blocking autophagy would result in a better functional outcome, especially in the long term. In humans, few data are available and only from the acute phase: Vanhorebeek et al. observed an accumulation of damaged organelles and autophagosomes in skeletal muscles and livers of ICU patients, suggesting insufficient autophagy [30]. Autophagy is a highly conserved process that recycles damaged or dysfunctional components through a lysosome‐dependent degradation, which maintains muscle homeostasis and function [21]. The conflicting results among studies of sepsis may be linked to the pertinence of the experimental model employed, but perhaps also to the methods used to study autophagy. As autophagy is a dynamic process, the current guidelines recommend monitoring autophagy flux, defined as a measure of autophagic degradation activity, typically assessed by blocking autophagosome‐lysosome fusion or lysosomal proteolysis [14]. Few data are available in the field of sepsis from in vivo models. Our study is the first to examine in vivo autophagy flux in the skeletal muscles of sepsis survivors. We developed a model to inhibit the autophagosome‐lysosome fusion step with CQ in sepsis mice and assessed autophagy using reference techniques, notably the turnover of LC3B‐II and P62 [14, 17]. Our protocol succeeded to the extent that SPF mice treated with CQ exhibited a measurable blockade of their autophagy flux, and we were thus able to demonstrate that autophagy flux was completely blocked in the muscle tissue (i.e., quadriceps and *tibialis anterior*) of sepsis survivors. That the CQ‐mediated effects did not worsen the muscle phenotype is likely because the flux was already fully inhibited. In accordance with our results, using a resuscitated caecal ligature puncture (CLP) model with caecal resection at Day 3, Crowell et al. reported a biological state similar to autophagy inhibition among sepsis survivors, as evidenced by unchanged AMPK phosphorylation, increased mTOR kinase activity and Ser757 phosphorylation of Ulk1, compared with pair‐fed controls [31]. Also, in line with our results, Dos Santos et al. assessed some autophagy‐related proteins in humans 6 months after critical illness and found that, compared with controls, there was an increase in BECLIN1, which is implicated in the initiation of autophagy. From our interrogations of their data, we also found that one of the 3 modules associated with muscle and physical dysfunction was related to mitochondrial dynamics (MitoCarta3.0), autophagosome and lysosome membrane (GO terms) in human ICU survivors. Importantly, we have also demonstrated that the impact of sepsis upon mitochondria and muscle can be prevented by enhancing the formation and degradation of autophagosomes using a pharmacological approach. This study shows that the sepsis‐induced blockade of autophagy flux is reversible, as we succeeded in stimulating it using UA. Others have shown that rapamycin, a known autophagy inducer, improved muscle function at 7 days post‐CLP and resuscitation [32]. Although our study demonstrates that the removal of damaged mitochondria is impaired due to defective autophagy, it does not dissect the specific molecular mechanisms involved. Sepsis survivors exhibited increased levels of PARKIN, PINK1 and BNIP3, suggesting impairment in both ubiquitin‐dependent and receptor‐mediated mitophagy pathways, but this was not thoroughly investigated. Further experiments would be required, such as the use of muscle‐specific knockout models targeting key mitophagy mediators (e.g., Parkin and Bnip3), or dedicated analyses of mitophagy flux using mitochondria‐targeted reporter probes (e.g., mito‐Keima and mito‐SRAI) [33, 34]. Although taking muscle biopsies of already frail patients raises significant ethical concerns in a translational research context, our work highlights the need to investigate mitochondrial function, dynamics and autophagy flux in the skeletal muscle of ICU survivors to investigate novel therapeutic options and address the limitations of current treatments.

Nutrition and physical rehabilitation are considered key pillars in the treatment of post‐ICU muscle weakness and physical disabilities [35, 36], but their optimization with a multidisciplinary protocol has not provided conclusive evidence of improved outcomes in comparison with standard care [35, 36, 37]. Indeed, ICU survivors may remain weak because of profound cellular abnormalities that persist in the long term even after restoring calorie balance [38, 39]. Targeting mitochondria through autophagy inducers could enhance the benefits of nutritional and exercise‐based interventions. Although rapamycin has been shown to regulate autophagy pathways in humans, notably by inhibiting mTOR, it also has a broad range of systemic influences including immunosuppressive and antiproliferative effects [40]. As ICU survivors are often already confronted with both immunosuppression and dysfunction of immune cells, these properties may limit its utility. Although discovered 40 years ago, UA, a natural metabolite derived from the metabolism of ellagitannins by gut microbiota, has recently garnered renewed research attention. It has been proven to be safe and effective in promoting mitochondrial health in humans [15]. UA has been reported to induce non‐selective autophagy [41, 42], to improve muscle function in muscular dystrophy [16] and to counteract muscle ageing in humans [15]. Nevertheless, little is known about the beneficial effects of UA in the field of sepsis. Here, we have provided a molecular basis for UA's prevention of muscle and mitochondrial dysfunction in sepsis survivors. UA improved mitochondrial and muscle quality rather than quantity. UA fully restored OXPHOS capacities without increasing mitochondrial biomass and induced a myofibre shift towards a more oxidative phenotype without modifying myofibre area. Notably, UA acts through the promotion of autophagy, as demonstrated by the reversal of mitochondrial and muscle improvements when autophagic flux was pharmacologically blocked, both in vivo and in vitro. Importantly, UA enhanced autophagy by simultaneously increasing the synthesis and degradation of autophagic vacuoles, thereby restoring autophagic flux. Collectively, these findings suggest that repurposing UA holds promise for enhancing long‐term patient outcomes following sepsis, particularly in muscle‐related conditions, making it a compelling candidate for future translational research.

## Ethics Statement

All authors certify in their manuscript that they comply with the Ethical guidelines for authorship and publishing in the *Journal of Cachexia, Sarcopenia and Muscle*.

## Conflicts of Interest

The authors declare no conflicts of interest.

## Supporting information


**Figure S1:** Transcriptomic analysis reveals sustained dysregulation of mitochondria‐related genes in the skeletal muscle of human ICU survivors.
**Figure S2:** Murine CSI‐induced sepsis model with resuscitation: changes in clinical characteristics and body composition over time.
**Figure S3:** Murine CSI‐induced sepsis model with resuscitation: muscle phenotype in survivors.
**Figure S4:** Murine CSI‐induced sepsis model with resuscitation: changes in systemic metabolism characteristics over time.
**Figure S5:** Murine CSI‐induced sepsis model with autophagy blockade.
**Figure S6:** Green synthesis of urolithin A: eco‐catalysed Hurtley reaction.
**Figure S7:** CSI‐induced sepsis model with urolithin A: clinical characteristics and muscle phenotype.
**Figure S8:** CSI‐induced sepsis model with urolithin A: autophagy flux.
**Figure S9:** Visual abstract.
**Table S1:** Description of the Murine Sepsis Score.
**Table S2:** Description of the antibodies used in western blot and Immunofluorescence experiments. jcsm70041‐sup‐0001‐Supplementary_Material.pdf.
**Table S3:** Description of the primers used in RT‐qPCR experiments (relative RNA expression).
**Table S4:** Description of the primers used in qPCR experiments (mitochondrial DNA copy number).
