# Supplementary material for: Sepsis Induces Long‐Term Muscle and Mitochondrial Dysfunction due to Autophagy Disruption Amenable by Urolithin A
Source: J Cachexia Sarcopenia Muscle. 2025 Aug 15;16(4):e70041. doi: 10.1002/jcsm.70041 (PMC12356704; doi:10.1002/jcsm.70041)
Supplement: Supplementary file 1 — Figure S1: Transcriptomic analysis reveals sustained dysregulation of mitochondria‐related genes in the skeletal muscle of human ICU survivors. Figure S2: Murine CSI‐induced sepsis model with resuscitation: changes in clinical characteristics and body composition over time. Figure S3: Murine CSI‐induced sepsis model with resuscitation: muscle phenotype in survivors. Figure S4: Murine CSI‐induced sepsis model with resuscitation: changes in systemic metabolism characteristics over time. Figure S5: Murine CSI‐induced sepsis model with autophagy blockade. Figure S6: Green synthesis of urolithin A: eco‐catalysed Hurtley reaction. Figure S7: CSI‐induced sepsis model with urolithin A: clinical characteristics and muscle phenotype. Figure S8: CSI‐induced sepsis model with urolithin A: autophagy flux. Figure S9: Visual abstract. Table S1: Description of the Murine Sepsis Score. Table S2: Description of the antibodies used in western blot and Immunofluorescence experiments. jcsm70041‐sup‐0001‐Supplementary_Material.pdf. Table S3: Description of the primers used in RT‐qPCR experiments (relative RNA expression). Table S4: Description of the primers used in qPCR experiments (mitochondrial DNA copy number). [file JCSM-16-e70041-s001.pdf]

Supporting Information

Detailed protocols and reagents

Supplementary figures

- Fig. S1. Transcriptomic analysis reveals sustained dysregulation of mitochondria-related genes in the skeletal muscle of human ICU survivors.
- Fig. S2. Murine CSI-induced sepsis model with resuscitation: changes in clinical characteristics and body composition over time.
- Fig. S3. Murine CSI-induced sepsis model with resuscitation: muscle phenotype in survivors.
- Fig. S4. Murine CSI-induced sepsis model with resuscitation: changes in systemic metabolism characteristics over time.
- Fig. S5. Murine CSI-induced sepsis model with autophagy blockade.
- Fig. S6. Green synthesis of Urolithin A: ecocatalysed Hurtley reaction.
- Fig. S7. CSI-induced sepsis model with urolithin A: clinical characteristics and muscle phenotype.
- Fig. S8. CSI-induced sepsis model with urolithin A: autophagy flux.
- Fig. S9. Visual abstract.

Supplementary tables

- Table S1. Description of the Murine Sepsis Score.
- Table S2. Description of the antibodies used in Western-blot and Immunofluorescence experiments.
- Table S3. Description of the primers used in RTqPCR experiments (relative RNA expression).
- Table S4. Description of the primers used in qPCR experiments (mitochondrial DNA copy number).

Whole western blots

Supplementary references

Detailed protocols and reagents

Bioinformatic analysis

Lists of differentially expressed genes (DEGs) for D7 vs CTRL and M6 vs CTRL contrasts as well as WGCNA gene modules were extracted from supplementary table and information from the study by Walsh *et al.* (1). Enrichment analyses on DEGs and gene modules were performed using R (v4.3.1) and dedicated R packages depending on the pathways under consideration. For the sake of comparison with Walsh *et al.*, gprofiler (v0.2.3) was used to scan GO:BP, GO:CC, GO:MF, KEGG and HP pathways. To scan MitoCarta3.0 Human pathways (2), clusterProfiler (v4.10.0) was used as it can integrate external pathway databases. Both methods were used similarly, using Over-Representation-Analysis (ORA), controlling FDR with the Benjamini–Hochberg procedure and considering the same set of genes as a background, genes which were identified following the methodology described in Walsh *et al.* using the GSE78929 dataset available in GEO data repository. Enrichment dotplots and barplots were generated with the R package ggplot2 (v3.5.1). Gene expression heatmaps were generated with the R package pheatmap (v1.0.12) using correlation as distance measure and ward.D2 for clustering. Muscle and physical dysfunction were defined by Walsh *et al.* as a reduced muscle mass (measured by quadriceps' cross sectional area), muscle strength (measured by MRC sum score or peak torque), and physical function (assessed by Functional Independence Measure) (1).

The murine sepsis model with delayed resuscitation

Male and female C57BL/6J mice aged 18 to 24 weeks old (Charles Rivers Laboratories, Saint Germain Nuelles, France) were housed in a dedicated, pathogen-free animal facility (21 °C, 12 h light/dark cycles) with free access to water and food (except for the mice in

the pair-feeding experiments). Sepsis was induced with an intraperitoneal (IP) injection of cecal slurry (CSI) solution (400 µL for male and 300 µL for female), prepared at a concentration of 100 mg of stool from a batch of donor mice per 1 mL of 10% glycerol–PBS, while sham mice were injected with a 10% glycerol–PBS IP injection (3–5). Experimental model refinement, human endpoints and bacteriological characteristics have been previously described (5). Sepsis mice were excluded from the study for 2 reasons: at the 12th hour post-CSI, if Murine Sepsis Score was below 10 and temperature above 30°C, mice were excluded as they were considered to have a non-severe infection rather than sepsis. At day 10 post-CSI, if peritoneal cavity examination revealed the presence of abscesses, mice were also excluded as they were not considered as sepsis survivors but rather as having a persistent chronic infection. (1) To assess the impact of the energy intake reduction on muscle phenotype, mice were divided into three groups: sham fed (SF), sham pair-fed (SPF), and sepsis. (2) To assess autophagy flux in skeletal muscle, mice were treated with chloroquine (CQ), which inhibits autophagy flux by reducing autophagosome-lysosome fusion (6), and divided into four groups: SPF vehicle (Ve), SPF CQ, Sepsis Ve and Sepsis CQ. (3) To assess whether increasing autophagic flux improved muscle phenotype, we used urolithin A (UA), a natural compound known to induce autophagy and mitophagy. Mice were divided into four groups: SPF placebo (Pl), SPF UA, Sepsis Pl and Sepsis UA. (4) To evaluate whether UA increased autophagy flux in our model, UA and CQ were concomitantly administered in SPF and Sepsis mice. All groups were resuscitated (NaCl 0.9% 25 mL/kg subcutaneously, imipenem–cilastatin 40 mg/kg IP and buprenorphine 0.1 mg/kg IP every 12 h for 5 days) from the 12th hour post-CSI to mimic human temporality. Intraperitoneal injections of CQ (first dose 30 mg/kg, then 60 mg/kg) began at the 12th hour and continued every 24 hours for 10 days, control

mice received Ve (200 µL of 0.9 % NaCl). Similarly, UA (15 mg/kg) was injected intraperitoneally from the 12th hour, every 12 hours until day 5, then every 24 hours until day 10; control mice received Pl (300 µL of 0.9% NaCl and 0.1% DMSO). The SF and sepsis mice had free access to food, while the SPF mice had only restricted access according to a pair-feeding procedure, receiving the same quantity of food consumed by the sepsis-surviving (SS) mice. Mice were individualized in cages to quantify food intake. If SS mice consumed more food than SPF mice, we did not force-feed the SPF mice to make up for the difference in food intake. Each SPF mouse was matched to the weight of an SS mouse (±2 g). Mice were randomized at 12 hours according to basal body weight and clinical severity parameters (MSS and temperature at the 12th hour) using the software RandoMice (7). Investigators were blinded during the physiological assessments and data analysis processes, but owing to its distinctive yellow color the administration of UA to animals was not blinded. Mice were monitored every 12 hours. Xiphoid surface temperature was measured using an infrared thermometer (Lasergrip 774, Etekcity, Anaheim, USA). The Murine Sepsis Score is a clinical severity score adapted to murine sepsis and assesses seven items scored from 0 to 4, where 0 is normal and 4 is the most severe grade (Table **S1**). Mice were euthanized by cervical dislocation at day 10 for muscle harvesting and assays. Physiological assessments of muscle and mitochondrial function were performed immediately after mouse sacrifice, with special attention being given to the timing of sacrifice, particularly for the autophagy experiments. All animal procedures were approved by the local ethics committee (Animal Experimentation Ethics CEEA75, Lille, France, APAFIS#18107-2018121214148408).

Body composition and indirect calorimetry

Body composition was assessed in unanesthetized mice using time-domain nuclear magnetic resonance (TD-NMR) (Minispec fl50 7.5 MHz, Bruker, Billerica, USA). Following a 72-hour acclimatization period, parameters including food intake, locomotor activity, CO_2 production (V_{CO2}), and O_2 consumption (V_{O2}) were quantified using an indirect calorimetry system (Phenomaster, TSE Systems, Berlin, Germany). Food consumption was measured by weight sensors attached to the top of food containers suspended in the cage. Ambulatory activity was measured by an infrared beam system covering the horizontal surface of the cage that continuously quantified the animal's movement. V_{CO2} and V_{O2} (mL/h) were continuously measured for 2 min per cage every 20 min and averaged over this period. The respiratory exchange ratio (RER) was computed as [V_{CO2}/V_{O2}], energy expenditure (kcal/h) as [(3.815 + 1.232 × RER) × V_{O2}) × 1000]. The daily energy intake (kcal/g/day) was determined based on the diet's caloric value, daily energy expenditure as [energy expenditure × 24], and daily energy balance as [daily energy intake – daily energy expenditure]. A negative energy balance represents an energy deficit. The diet (U8220G10R, SAFE, France) contained 3,3 kcal/g food, 19.3% proteins, 8.4% fat, and 72.4% carbohydrate. All these measurements were normalized to body weight.

Green synthesis of 3,8-dihydroxy-6H-benzo[c]chromen-6-one (urolithin A)

The synthesis of the title compound urolithin A was accomplished by a green Hurtley reaction starting from resorcinol, a bio-sourced reagent (8). First, the commercial 2-bromo-5-hydroxybenzoic acid (10.0 g, 1 equiv.) was stirred in aqueous sodium hydroxide (3.69 g, 2 equiv.) with resorcinol (10.15g, 2 equiv.) for 30 min at 50 °C. The resulting mixture was then heated to 100 °C for 3 hours in presence of catalytic amount of Ecocat-MG2 obtained from Miscanthus x giganteus1 (0.06 equiv., 2% mol of metals) or copper sulfate

(0.016 equiv.). After cooling to rt, the medium was concentrated in vacuo and ethanol was then added to the crude. The precipitate formed was filtered, washed with absolute ethanol and recrystallized from absolute ethanol to provide pure urolithin A in 78% yield (8.2 g) as a light-yellow solid. Starting materials are commercially available and were used without further purification (suppliers: TCI Europe N. V. and Carlo Erba Reagents S.A.S.) except for ecocatalyst Ecocat-MG2 which was produced by our group from ryegrass cultivated on contaminated soils following our described procedure. Nuclear magnetic resonance (NMR) spectra were acquired at 400 MHz for 1H NMR, and at 100 MHz for 13C NMR on a Varian 400-MR spectrometer with tetramethylsilane (TMS) as internal standard, at room temperature (RT). Chemical shifts (δ) are expressed in ppm relative to TMS. Splitting patterns are designed: s, singlet; d, doublet; br s, broaden singlet. Coupling constants (J) are reported in Hertz (Hz). ^{1}H NMR (DMSO-d_6, 400 MHz) δ ppm 6.73 (s, 1H, Ar*H*), 6.81 (d, *J* = 7.7 Hz, 1H, Ar*H*), 7.32 (d, *J* = 7.7 Hz, 1H, Ar*H*), 7.51 (s, 1H, Ar*H*), 8.03 (d, *J* = 7.4 Hz, 1H, Ar*H*), 8.12 (d, *J* = 7.4 Hz, 1H, Ar*H*), 10.1 (br s, 1H, O*H*), 10.2 (br s, 1H, Ar*H*); ^{13}C NMR (DMSO-d_6, 100 MHz) 102.7 (CH), 109.7 (C), 112.9 (CH), 113.4 (CH), 120.0 (C), 123.5 (CH), 123.7 (CH), 124.1 (CH), 126.8 (C), 150.8 (C), 156.8 (C), 158.4 (C), 160.5 (C).

Mitochondrial respiration of permeabilized muscle fibers

The oxygen consumption (J_{O2}) of the *Soleus* and *Extensor Digitorum Longus* (EDL) pmf was assessed with High-Resolution Respirometry (Oroboros Instruments, Innsbruck, Austria). *Soleus* and *Extensor Digitorum Longus* (EDL) muscles were mechanically separated in a cold BIOPS solution, then permeabilized (using 50 µg/L saponin in BIOPS with gentle agitation at 4 °C for 30 and 20 minutes, respectively). Subsequently, the muscle fibers were rinsed twice in the mitochondrial respiration medium Mir05 and weighed on a

precision balance (CPA225D, Sartorius, Göttingen, Germany) (9). The J_{O2} of the muscle fibers was standardized to their wet weight, assessed at 25 °C utilizing the O2K and recorded with DatLab 7.4 software: 2–4 mg of fibers were placed in each chamber containing 2 mL of oxygenated Mir05 (O_2 concentration close to 400 nmol/L). Mitochondrial substrates and inhibitors were successively introduced after achieving a steady state, following two procedures (10). In the protocol assessing carbohydrate oxidation, the injection of pyruvate (5 mM), malate (2 mM), and glutamate (10 mM) without ADP assessed the J_{O2} of the electron transport chain (ETC) uncoupled from ATP synthase (denoted PMG). The injection of ADP (5 mM) evaluated the J_{O2} of the ETC coupled with ATP synthase (OXPHOS state) and primarily driven by complex I (denoted CI-CIV). Inhibiting complex I with rotenone (0.5 µM) and adding succinate (10 mM, a complex II substrate) assessed the OXPHOS J_{O2} driven by complex II (denoted CII-CIV). In the protocol assessing fatty acid oxidation, adding octanoyl-L-carnitine (0.5 mM) and malate (2 mM) (denoted OCT-M) followed by ADP (5 mM) evaluated the OXPHOS state preferentially driven by complexes I and II (denoted CI + II-IV). Quality control was implemented for each sample: where J_{O2} increased by +15% after cytochrome c (10 µM) addition in the OXPHOS state, samples were excluded from analysis as this indicated mitochondrial damage had occurred during permeabilization.

Mitochondrial respiration of isolated mitochondria

The oxygen consumption rate (OCR) of the *Tibialis anterior* was assessed with the Seahorse XFe96 Analyzer (Agilent; Les Ulis, France). *Tibialis anterior* was minced into small pieces with micro-scissors in ice-cold Mitochondrial Isolation Buffer (MIB: 300 mM sucrose, 5 mM TES, 0.2 mM EGTA, pH 7.2), then homogenised by 8 passes of a loose

pestle in a Dounce cylinder. Centrifugation at 800 g (10 minutes, 4°C) was performed, and the supernatant aspirated before further centrifugation at 8000 g (10 minutes, 4°C) to obtain a mitochondria pellet, which was solubilized in MIB. Bradford protein assays were performed according to the manufacturer's instructions. Isolated mitochondria were aliquoted into the Seahorse cell plate at a concentration of 5 µg per well (50 µL / well). The plate was centrifuged at 2000 g for 20 minutes at 4°C to ensure even distribution of mitochondria. After centrifugation, 100 µL of pre-warmed Mitochondrial Assay Solution (MAS: mannitol 220 mM final, sucrose 70 mM, KH_2PO_4 10 mM, $MgCl_2$ 5 mM, HEPES 2 mM, EGTA 1 mM, and 0.2% fatty acid-free BSA) was added to each well, and the plate was incubated at 37°C for 10 minutes. The OCR was recorded in real-time, with substrates and inhibitors to assess the mitochondrial respiratory chain activity. Substrates included pyruvate, malate, glutamate (10 mM each), then ADP (5 mM), rotenone (2 µM) and antimycin A (4 µM), then TMPD (100 µM), ascorbate (10 mM) and sodium azide (15 mM). Specific steps were followed to ensure accurate preparation of each reagent and proper loading into the Seahorse cartridge.

***In situ* contractility**

Mice were deeply anesthetized with an intraperitoneal injection of ketamine (20 mg/kg) followed by continuous isoflurane inhalation. The dissection and measurement protocol has been previously described (11). In summary, the right hind limb's muscles, excluding the *Soleus* muscle, were subjected to denervation, and the *Soleus* muscle itself was carefully separated from surrounding tissues. Subsequently, the limb was placed in a paraffin oil bath, maintained at a constant temperature of 37°C, and fixed using bars and pins. The *Soleus* muscle was positioned horizontally, and its distal tendon attached to a

force transducer (Grass FT 10, Grass Instruments). The muscle length was adjusted to achieve maximum twitch peak tension. Contraction was induced by stimulating the sciatic nerve with 0.2 ms pulses through bipolar platinum electrodes, using twice the minimum voltage necessary for eliciting the maximal twitch response. The first protocol determined the force-frequency curve and the maximal force of the *Soleus* by tetanic stimulations with increasing frequency (16 to 100 Hz). The second assessed the fatigability of the *Soleus* by repeated tetanic stimulations at 40 Hz for 120 seconds. Finally, the muscle was removed to determine its wet weight. Muscle force was normalized to muscle wet weight. The experiments were conducted in a blinded manner.

***Ex vivo* contractility**

Soleus and EDL muscles were dissected under continuous irrigation with Krebs solution (120 mM NaCl, 4.7 mM KCl, 1.25 mM $CaCl_2$, 1.2 mM $MgSO_4$, 25 mM $NaHCO_3$, 1.2 mM KH_2PO_4, 11 mM D-glucose, and 2 mM Na-pyruvate). Following dissection, muscles were incubated at 25°C in the myograph bath containing the same Krebs solution continuously oxygenated with 95% O_2/5% CO_2, maintained at pH 7.4, and subsequently mounted by securing tendons to hooks within a DMT Muscle Strip Myograph system (model 820MO) coupled with a CS4+ stimulator. Muscles were equilibrated at optimal length (L_0), determined by adjusting length to achieve maximal twitch force at 1 Hz stimulation. Contractile parameters were recorded using an automated protocol (MyoPULSE software). Force-frequency relationships were established by stimulating muscles sequentially at frequencies of 15, 30, 50, 80, 100, 120, 140, 160, and 200 Hz. Following experiment, muscles were weighed following ligature removal, allowing calculation of physiological cross-sectional area for normalization of contractile force data.

Autophagy flux

Autophagy assessment was performed using a two-step model to analyze autophagosome formation and degradation using LC3-II turnover assays according to Plaza-Zabala *et al.* (12). Autophagy flux was blocked using the lysosomal inhibitor CQ, both *in vitro* and *in vivo*. The relative amount of LC3-II was measured by Western blot to assess the autophagosome pool. The autophagosome pool in control samples without lysosomal inhibitor represented the steady-state autophagosome pool. Autophagosome turnover in a steady-state was considered at equilibrium, meaning autophagosome formation was equal to the autophagosome degradation. Autophagosome formation under these experimental conditions was calculated by subtracting the steady-state autophagosome pool from the experimental autophagosome pool in the presence of lysosomal inhibitors. Autophagosome degradation was determined by the difference in the autophagosome pool with and without lysosomal inhibitors in both the steady state and experimental conditions. The degradation and formation ratios were obtained by dividing the degraded and formed autophagosome pools in the experimental condition by those in the steady state. The turnover ratio was represented by the ratio between the degradation and formation ratios.

First step:

- *Autophagosome formation (steady state) = autophagosome degradation (steady state)*
- *Autophagosome formation (experimental condition) =* $LC3B\text{-}II^{Exp+CQ+} - LC3B\text{-}II^{Exp-CQ-}$

- *Autophagosome degradation (steady state) =* $LC3B\text{-}II^{Exp\text{-}CQ+} - LC3B\text{-}II^{Exp\text{-}CQ-}$

- *Autophagosome degradation (experimental condition) =* $LC3B\text{-}II^{Exp+CQ+} - LC3B\text{-}II^{Exp+CQ-}$

Second step:

- *Degradation ratio =* $\frac{autophagosome\ degradation\ (experimental\ condition)}{autophagosome\ degradation\ (steady\ state)}$

- *Formation ratio =* $\frac{autophagosome\ formation\ (experimental\ condition)}{autophagosome\ formation\ (steady\ state)}$

- *Turnover ratio =* $\frac{degradation\ ratio}{formation\ ratio}$

Relative protein expression

Quadriceps or *Soleus* muscles were homogenized in 60 µL/mg of ice-cold RIPA buffer (containing 10 mM Tris–HCl pH 7.4, 5 mM EDTA, 0.1% SDS, 150 mM NaCl, 1% sodium deoxycholate, and 1% Triton), supplemented with 1 mM phenylmethanesulfonyl fluoride and a protease/phosphatase inhibitor cocktail (#5872, Cell Signaling, Danvers, USA), using a Bead Mill 4 Homogenizer (Thermo Fisher Scientific, Waltham, USA). Subsequently, the homogenates were centrifuged at 15,000× g for 10 minutes at 4 °C. The protein concentration was determined using Bradford Reagent (Sigma, Burlington, USA). Denatured proteins (40 µg) were separated by SDS polyacrylamide gel electrophoresis and transferred onto polyvinylidene fluoride membranes following the manufacturer's instructions (Invitrogen, Waltham, USA). After overnight incubation with primary antibodies and a 1-hour incubation with horseradish peroxidase-conjugated secondary

antibodies (Table S2), protein complexes were visualized using chemiluminescence (Kit Clarity Western ECL substrate, Bio-Rad, Hercules, USA) with the Fusion X Spectra system (Vilber, Marne-la-Vallée, France). Protein expression levels were quantified using ImageJ software (NIH, Bethesda, MD, USA) and normalized to actin or glyceraldehyde 3-phosphate dehydrogenase (GAPDH).

Biotin switch assay and pull down

Sixty-six milligrams of quadriceps muscle tissue was homogenized in 1 mL of HENS buffer (100 mM HEPES-NaOH, pH 8.0, 1 mM EDTA, 0.1 mM Neocuproine, and 1% SDS (w/v)) using a mechanical homogenizer. The homogenate was centrifuged at 15,000 g for 10 minutes at 4°C to remove insoluble material, and the supernatant was collected for further analysis. Protein concentration was determined using the Bradford protein assay. The biotin switch assay was performed according to the manufacturer's instructions (Cayman Chemical S-Nitrosylated Protein Detection Kit, Item No. 10006518), based on the method initially described by Jaffrey *et al.* (13), with modifications by Forrester *et al.* (14). Free thiols in the muscle protein extracts were blocked by incubating with 20 mM S-methyl methanethiosulfonate (MMTS) in HENS buffer at 50°C for 20 minutes in the dark. The reaction mixture was then precipitated and incubated with ice-cold acetone at −20 °C for 1 h, centrifuged at 3000 g for 10 min at 4°C, and gently washed three times with 70% acetone to remove excess MMTS. Protein pellets were resuspended and incubated in HENS buffer with ascorbate in the presence of biotin-HPDP (*N*-[6-(Biotinamido)hexyl]-3'-(2'-pyridyldithio)propionamide) at room temperature for 1 hour in the dark. This step selectively reduces S-nitrosothiols to thiols, which are then biotinylated. Proteins were again precipitated with acetone and washed three times with 70% acetone to remove

unreacted biotin-HPDP and other reagents. The resulting protein pellets were resuspended in 1:4 HENS/10 buffer (10 mM HENS, 0.1 mM EDTA, neocuproine 0.01 mM, SDS 1%, pH 8.0) and 3:4 neutralization buffer (25 mM HEPES, 100 mM NaCl, 0.5% triton, pH 7.5). Biotinylated proteins were pulled down by overnight incubation at 4°C with streptavidin-agarose beads (Thermo Fisher Scientific, 20357). The beads were washed extensively with wash buffer (25 mM HEPES, 600 mM NaCl, 0.5% triton, pH 7.5) to remove non-specifically bound proteins. Biotinylated proteins were eluted from the beads by incubation with elution buffer (HENS/10 buffer with Laemmli buffer 4X) at 95°C for 5 minutes. Input and biotinylated proteins were analyzed by Western blot.

Relative mRNA expression

Frozen quadriceps muscles were homogenized in TRIzol solution (Total RNA Isolation, Invitrogen, Life Technologies, USA) and the subsequent RNA extraction followed the instructions provided by the manufacturer. DNA was removed by treating with DNAse (DNase I, RNase-free, Thermo Fisher Scientific, USA) at 37 °C for 30 minutes, followed by inactivation with EDTA at 65 °C for 10 minutes using a thermocycler (MJ Mini Thermal Cycler, Bio-Rad, USA). Total RNA (1 µg) underwent reverse transcription using the High-Capacity cDNA Reverse Transcriptase Kit (Thermo Fisher Scientific, USA) in a thermocycler with temperature steps of 25 °C for 10 minutes, 37 °C for 2 hours, and 85 °C for 5 minutes. Real-time detection of the cDNA involved amplification utilizing the PowerUp SYBR Green Master Mix kit (Thermo Fisher Scientific, USA) on a QuantStudio 3 Real-Time PCR System sequencer (Applied Biosystems, Foster City, USA). The primers are detailed in Table S3. PCR cycles adhered to the manufacturer's instructions. Subsequent

data analysis employed the Quant Studio Design and Analysis Software (Thermo Fisher Scientific, USA), and relative quantification was carried out following the 2-ΔΔCt method.

Mitochondrial DNA copy number

The NADH dehydrogenase 1 (Nd1) and Nd2 genes, encoded by mitochondrial DNA (mtDNA) and known for their infrequent deletions, along with the nuclear DNA-encoded Peptidyl-prolyl cis-trans isomerase (Ppia) gene, a housekeeping gene, were subjected to qPCR amplification using the primers provided in Table S4. Quadriceps total DNA was extracted using the QIAamp Fast DNA tissue kit (QIAGEN, Hilden, Germany), following the manufacturer's instructions. The purity and concentration of the DNA were assessed using Nanodrop (Thermo Fisher Scientific, USA). Real-time qPCR assays were used to assay DNA (200 pg), as detailed above. The relative quantification of mtDNA normalized to nuclear Ppia was conducted using the 2-ΔΔCt method.

Muscle mass and myofibers cross-sectional area and typing

The wet weight of each muscle was measured using a precision balance (ME104, Mettler Toledo). The cross-sectional area (CSA) and the Myosin heavy chain (Myhc) fiber typing of the muscle fibers were measured by laminin-α2 and Myhc immunofluorescence labeling. Isopentane-frozen muscle, fixed to a cryosection support using OCT Embedding Matrix (CellPath, UK), was transversely cross-sectioned by 10 μm using a cryostat at −20 °C (CM3050 S, Leica, Wetzlar, Germany) and then placed on slides (Superfrost Plus, Thermo Scientific, USA). Muscle sections were washed and permeabilized (0.05% PBS–Triton for 5 min, three times, at room temperature (RT)) and then blocked (3% PBS-BSA for 1 h at RT). Each section was incubated with primary antibodies (dissolved in 1% PBS-BSA) overnight at 4°C and then secondary antibodies for 1 hour at RT (Table S2). The slide was

mounted on a coverslip (Diamant Star, Menzel-Gläser, Braunschweig, Germany) using Vectashield anti-fading medium (Vector Labs, Berlingame, USA). At least three whole muscle sections were imaged in mosaic for each sample by an automated slide scanner (Axioscan Z1, Zeiss, Oberkochen, Germany). Myhc typing of *Soleus* was analysed with Cell Counter using ImageJ. Feret's diameter, CSA and Myhc typing of *Tibilias anterior* were analysed with MuscleJ using ImageJ after an automatic quality analysis of the myofiber images (15).

Mitochondrial DNA oxidation

Mitochondrial DNA is more prone to oxidative damage than nuclear DNA due to its proximity with the ETC and its absence of efficient repair system. Oxo-guanine localized within the mitochondria reflects mitochondrial DNA oxidation. Permeabilized and blocked muscles sections (described above) were incubated with 8-oxoguanine and VDAC antibodies overnight at 4°C then secondary antibodies and 40,6-diamidino-2-phenylindole (DAPI) for 1 hour at RT (Table S2). Images were acquired with Confocal Spinning Disk and proceed in 3D with Imaris (Oxford Instruments, UK). Nucleus was reconstructed as an object and subtracted from the analysis. Colocalization was assessed by Manders B coefficient, which measures the fraction of fluorescence (i.e. intensity of voxel) in channel B (VDAC) that colocalizes with channel A (8-oxoG), normalized by the total fluorescence in channel B. It ranges from 0 to 1, where 0 indicates no colocalization and 1 perfect colocalization. Physiologically, it represents the proportion of mitochondria with oxidized DNA (16).

Transmission electron microscopy

Tibialis anterior muscles were fixed with 1% glutaraldehyde and 4% paraformaldehyde in 0.1 M sodium cacodylate, pH 6.8 buffer, at 4°C, minimum overnight. Muscles were cut into small, parallelepiped pieces in the muscle fiber orientation for further longitudinal cutting. Samples were post-fixed with 1% osmium tetroxide and 1.5% potassium ferricyanide, then with 1% uranyl acetate, all in distilled water at room temperature in the dark, for 1 hour. After washing, samples were dehydrated with solutions of increasing ethanol concentration. Samples were finally infiltrated with epoxy resin and cured at 60°C for 24 hours. Sections of 70-80 nm thickness, cut longitudinally, were deposited on formvar-coated grids and observed at 80 kV with a Hitachi H7500 TEM (Milexia, France). Images were acquired with a 1 Mpixel digital camera from AMT (Milexia, France). Mitochondria and autophagic structures were analyzed with ImageJ. A minimum of three fields of view (FOV) with a magnification of 10,000x were analyzed to measure mitochondrial content. At least five FOV with a magnification higher than 30,000x were observed per sample to assess mitochondrial damage and the number of autophagic vacuoles. The image analysis was conducted in a blinded manner.

Cell culture

C2C12 myoblast cells were obtained from ATCC (CRL-1772) and cultured in Dulbecco's Modified Eagle Medium high glucose (DMEM, Gibco, 41965-039) supplemented with 10% fetal bovine serum (FBS, Dutscher, SV30160.03) and 1% penicillin-streptomycin (P/S, Gibco, 15070-063). Cells were maintained at 37°C in a humidified atmosphere of 5% CO_2 and passaged at 70-80% confluence using 0.05% trypsin-EDTA (Fischer, 11580626). Upon reaching 80-90% confluence, the growth medium was replaced with differentiation medium consisting of DMEM supplemented with 1% horse serum (HS, Dutscher, S0910-

500) and 1% P/S. Differentiation medium was changed every 48 hours for 7 days, to ensure proper differentiation into myotubes. To assess the autophagy flux *in vitro*, differentiated C2C12 cells were treated overnight with lipopolysaccharide (LPS, Sigma, L2880-100) at a concentration of 500 ng/mL, chloroquine 50 µM (Thermo Scientific, 15368865), and urolithin A 50 µM (as previously described). To assess mitochondrial ROS production *in vitro*, differentiated C2C12 cells were treated 48 hours with LPS 500 ng/mL, adenosine triphosphate 1 mM (ATP, Sigma-Aldrich, A2383), chloroquine 50 µM and urolithin A 50 µM.

Flow cytometry

Cell viability was determined with a spectrometer (Fluidlab R300, Anvajo, Germany). Mitochondrial mass and ROS production in differentiated C2C12 cells were determined using MitoTracker Green FM (Thermo Fisher Scientific, M7514) and MitoSOX Red (Thermo Fisher Scientific, M36008). Cells were harvested and washed twice with PBS. Approximately $3x10^5$ cells were resuspended in 1 mL of pre-warmed PBS containing 100 nM MitoTracker Green FM and 5 µM MitoSOX Red, and incubated at 37°C for 30 minutes in the dark. Flow cytometric analysis was performed using CytoflexLX flow cytometer (Beckman Coulter). Forward and side scatter were used to exclude debris and dead cells from the analysis. Data acquisition was set to collect at least 10,000 events per sample. Compensation controls were included for both MitoTracker Green and MitoSOX Red to correct for spectral overlap. Single-stained control samples were used to set up the compensation matrix, and unstained cells served as negative controls. Flow cytometry data were analyzed using FlowJo software (BD Biosciences). Gating strategies were applied to identify live, single cells and to exclude doublets and debris. The mean

fluorescence intensity (MFI) for MitoTracker Green and MitoSOX Red was quantified for each sample.

Statistical analysis

All statistical analyses were performed with GraphPad Prism 10 (GraphPad, San Diego, USA). The quantitative values are expressed as mean values ± standard error of the mean (SEM). Kaplan–Meier survival curves were compared with the log-rank test. If the continuous values followed a normal distribution (Shapiro–Wilk test) and had sufficient number of individuals, comparisons between two groups were performed with two-tailed Student t-test, otherwise with Mann-Whitney test. Comparisons between three or more groups were performed with one-way ANOVA and, if statistically significant, by a post hoc Fisher's LSD or Tukey test. If standard deviations were not equal, comparisons between three or more groups were performed with Welch's ANOVA. Otherwise, the groups were compared with the non-parametric Kruskal–Wallis test and, if statistically significant, by a post-hoc Dunn's test. Comparisons of three or more groups over time were performed with a two-way ANOVA test with correction using the post-hoc Tukey test. Comparisons with a p value < 0.05 were considered statistically significant.

Supplementary Figures

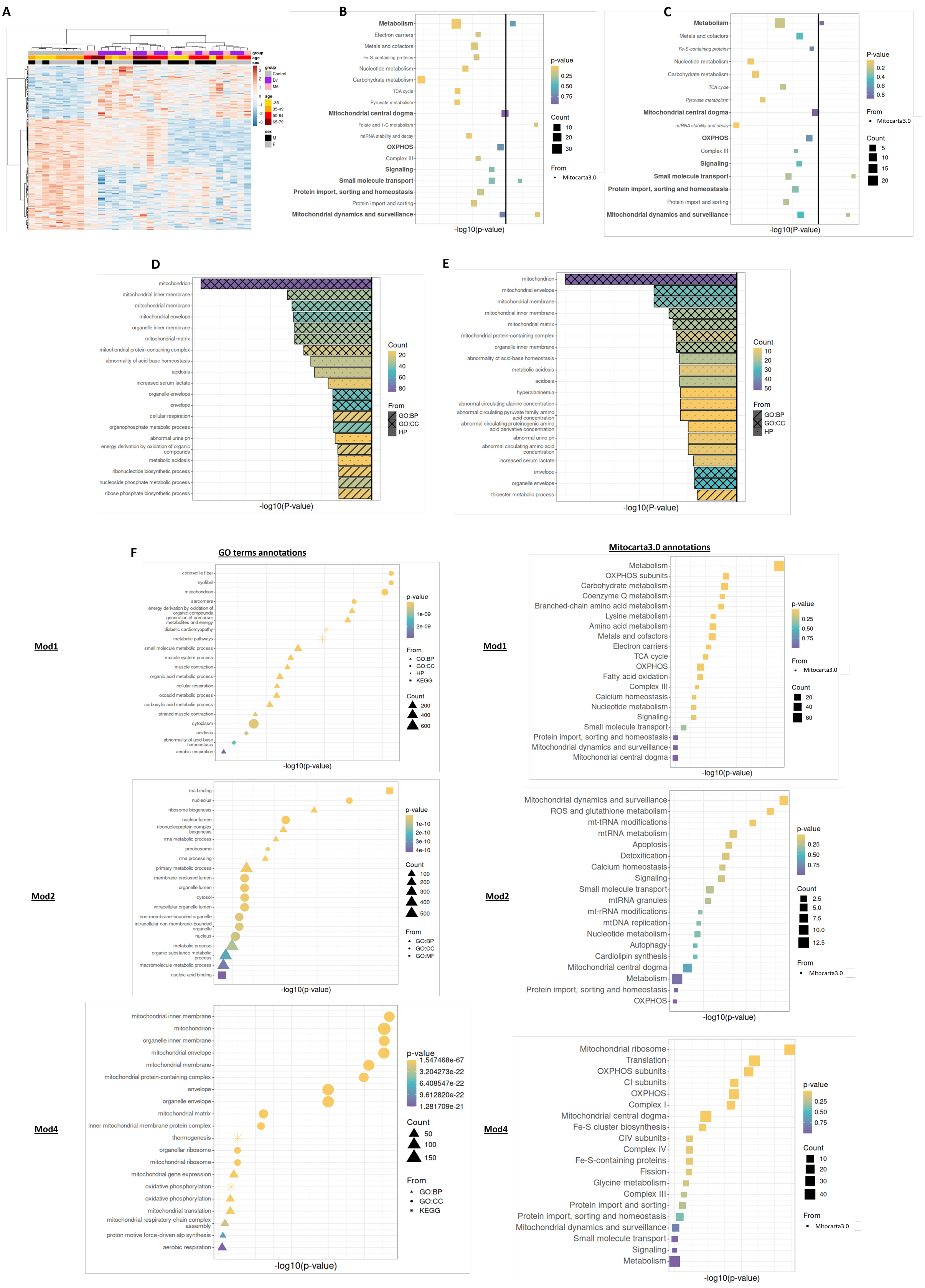

Figure S1. Transcriptomic analysis reveals sustained dysregulation of mitochondria-related genes in the skeletal muscle of human ICU survivors. A, Expression heatmap

of DEGs with an adjusted *p* value < 0.05 and an absolute (FC) > 1.5 in D7 *vs.* CTRL or M6 *vs.* CTRL. Rows and columns are clustered according to expression values. Additional annotations of samples (age, sex, group) are displayed. **B-C**, Dot plot representing the differentially expressed gens (DEGs) among the MitoCarta3.0 pathway enrichment analysis of D7 *vs.* CTRL (**B**) and M6 *vs.* CTRL (**C**). Enrichments over down-regulated genes are shown on the left side of the vertical solid line, while enrichments over up-regulated genes are shown on the right side. The gradient color indicates the adjusted *p* value of the enrichment. The principal mito-pathways are in bold font and the top 10 sub mito-pathways are in non-bold font. **D-E**, Bar plot representing the top-20 enriched pathways in D7 *vs.* CTRL DEGs among GO:BP, GO:CC, GO:MF, KEGG and HP sets (**D**) and in M6 *vs.* CTRL (**E**); bars indicate the GO category of the pathway and the color gradient the DEGs count. **F**, Dot plots representing the top-20 enriched pathways in modules 1, 2 and 4 of WGCNA using GO:BP, GO:CC, GO:MF, KEGG and HP sets (left) and Human MitoCarta3.0 (right). GO, Gene Ontology; BP, Biological Process; CC, Cellular Component; MF, Molecular Function; KEEG, Kyoto Encyclopedia of Genes and Genomes; HP, Human Phenotype; WGCNA, Weighted Gene Co-expression Network Analysis.

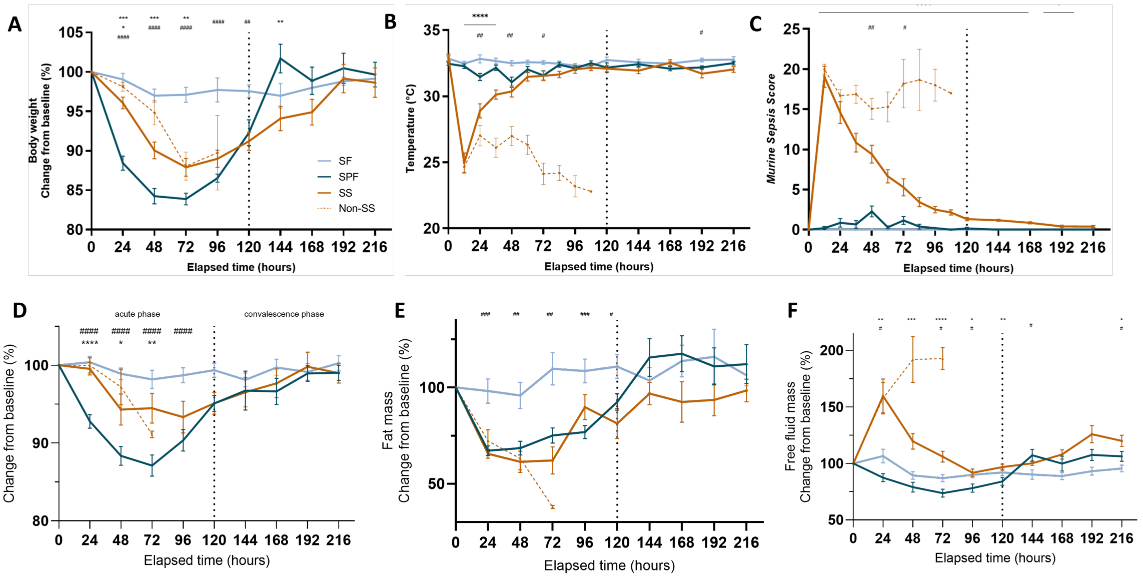

Figure S2. Murine CSI-induced sepsis model with resuscitation: changes in clinical characteristics and body composition over time. A-C, Body weight change from baseline (**A**), xiphoid surface temperature using infrared thermometer (**B**), Murine Sepsis Score (**C**) over time for SF mice (n = 18), SPF mice (n = 15), SS mice (n = 20), non-SS mice (n = 19). **D-F**, Changes from baseline over time in lean mass (**D**), fat mass (**E**) and free fluid mass (**F**) for SF mice (n = 14), SPF mice (n = 15), SS mice (n = 8), non-SS mice (n = 7). SF, sham fed mice in light blue; SPF, sham pair-fed mice in dark blue; SS, sepsis-surviving mice in orange; non-SS, non-surviving sepsis mice in dotted orange. Statistical comparison between SPF *vs.* SF (#) and SS *vs.* SPF (*), no comparison for the non-SS

group. Data analyzed with two-way ANOVA test. * p < 0.05, ** p < 0.01, *** p < 0.001, **** p < 0.0001.

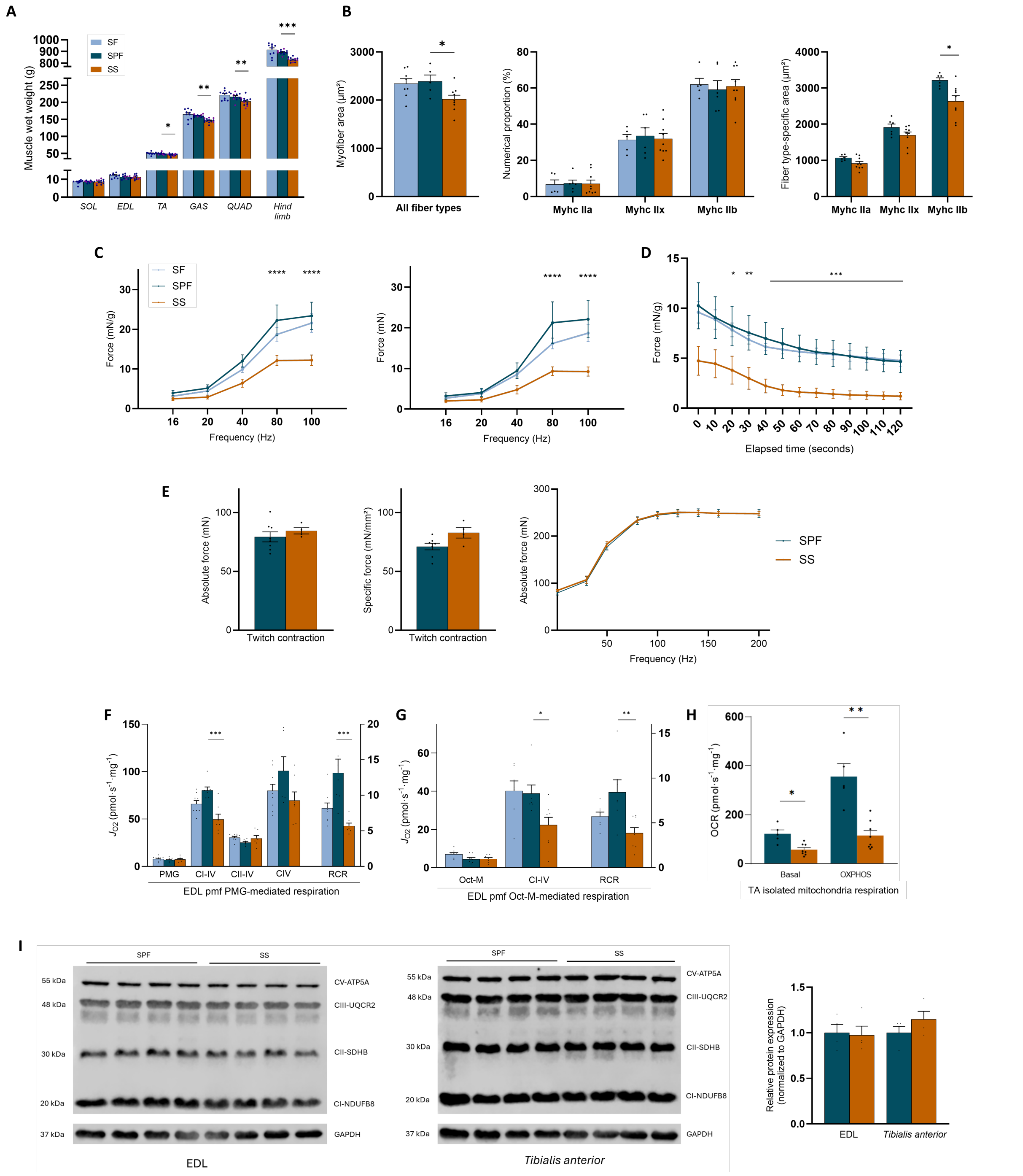

Figure S3. Murine CSI-induced sepsis model with resuscitation: muscle phenotype in survivors. A, Muscle wet weight. *SOL*, *Soleus*, *EDL*, *Extensor Digitorum Longus*, *TA*, *Tibialis Anterior*, *GAS*, *Gastrocnemius*, *QUAD,* quadriceps. The term "hind limb" refers to the sum of the *SOL*, *EDL*, *TA*, *GAS*, and *QUAD* of both limbs from SF mice (n = 10), SPF mice (n = 7 males and n = 4 females), SS mice (n = 9 males and n = 4 females). **B**, *Tibialis anterior* global CSA (left), fiber type specific CSA (middle) and Myhc type proportion (right)

for SF mice (n = 8), SPF mice (n = 6), SS mice (n = 9). **C**, *In situ* contractility of Soleus: force frequency curve determined by tetanic electrical stimulation of the sciatic nerve in anesthetized SF mice (n = 8), SPF mice (n = 6 males and n = 4 females), SS mice (n = 8 males and n = 3 females). Normalized (left) and non-normalized forces (right) forces. **D**, *Soleus* endurance force of *in situ* contractility experiments for SF mice (n = 8), SPF mice (n = 6), SS mice (n = 8). **E**, Ex vivo contractility of EDL: absolute twitch contraction (left), specific twitch contraction (middle), absolute force frequency curve (right) (n = 4 – 8 for each group). **F-G**, PMG-linked EDL pmf J_{O2} (**F**) and Octanoyl-carnitine-malate linked EDL pmf J_{O2} (**G**) (n = 7 – 10 for each group). The respiratory control ratio (RCR) is plotted on the right Y-axis in each graph. One value (SF) and 2 values (SS) excluded due to elevation of cytochrome c. **H**, Oxygen consumption rates (OCR) using Metabolic Flux Analyzer in isolated mitochondria of *Tibialis anterior* with pyruvate, malate, glutamate (10 mM each) then ADP (5 mM) for SPF mice (n = 5) and SS mice (n = 8). **I**, Protein expression of respiratory chain subunits in the EDL (left) and Tibialis anterior (right) (n = 4 for each group). SF, sham fed mice in light blue; SPF, sham pair-fed mice in dark blue; SS, sepsis-surviving mice in orange; non-SS, non-surviving sepsis mice in dotted orange. Statistical comparison between SPF *vs.* SF (#) and SS *vs.* SPF (*), no comparison for the non-SS group. Data analyzed with two-way ANOVA test (**C-E**), Kruskal Wallis test with post-hoc Dunn's test (**A-B**, **F**, **G**, **I**), Mann-Whitney test (**E**, **H**). * p < 0.05, ** p < 0.01, *** p < 0.001, **** p < 0.0001.

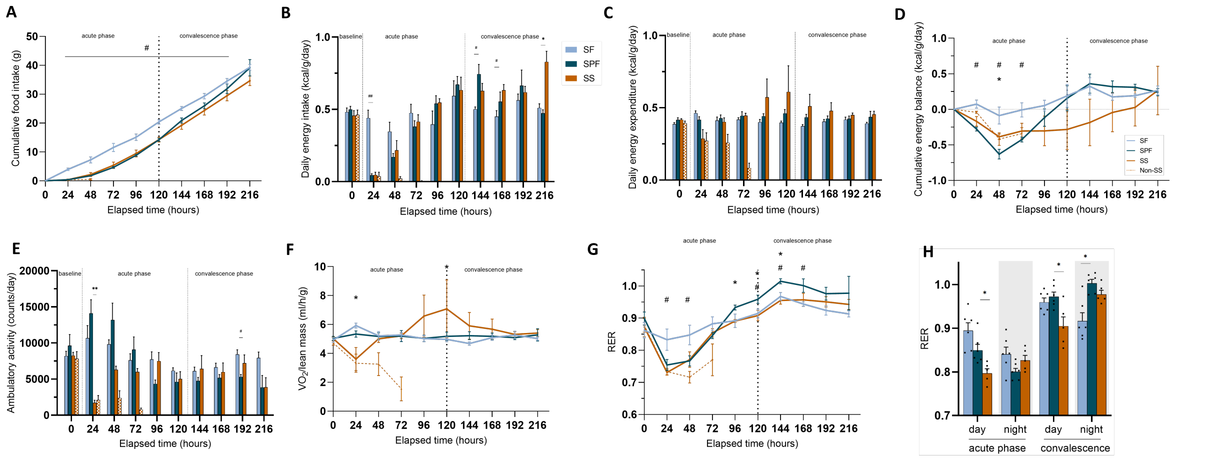

Figure S4. Murine CSI-induced sepsis model with resuscitation: changes in systemic metabolism characteristics over time. Indirect calorimetry in metabolic chamber after acclimation. Time 0 represents the day before the i.p. injection of cecal slurry or PBS–glycerol (baseline). Cumulative food intake (**A**), daily energy intake (**B**), daily energy expenditure (**C**), cumulative energy balance, as the cumulative energy intake minus expenditure (**D**), daily ambulatory activity (**E**) and daily systemic oxygen consumption (V_{O2}) normalized to lean mass (**F**), and Respiratory Exchange Ratio (RER) (**G**) over time. RER plotted by acute or convalescent phase and by day and night (**H**). SF mice (n = 6), SPF

mice (n = 6), SS mice (n = 5), non-SS mice (n = 5). SF, sham fed mice in light blue; SPF, sham pair-fed mice in dark blue; SS, sepsis-surviving mice in orange; non-SS, non-surviving sepsis mice in dotted orange. Statistical comparison between SPF *vs.* SF (#) and SS *vs.* SPF (*), no comparison for the non-SS group. Data analyzed with two-way ANOVA test (**A-G**), Kruskal Wallis test with post-hoc Dunn's test (**H**). * p < 0.05, ** p < 0.01, *** p < 0.001, **** p < 0.0001.

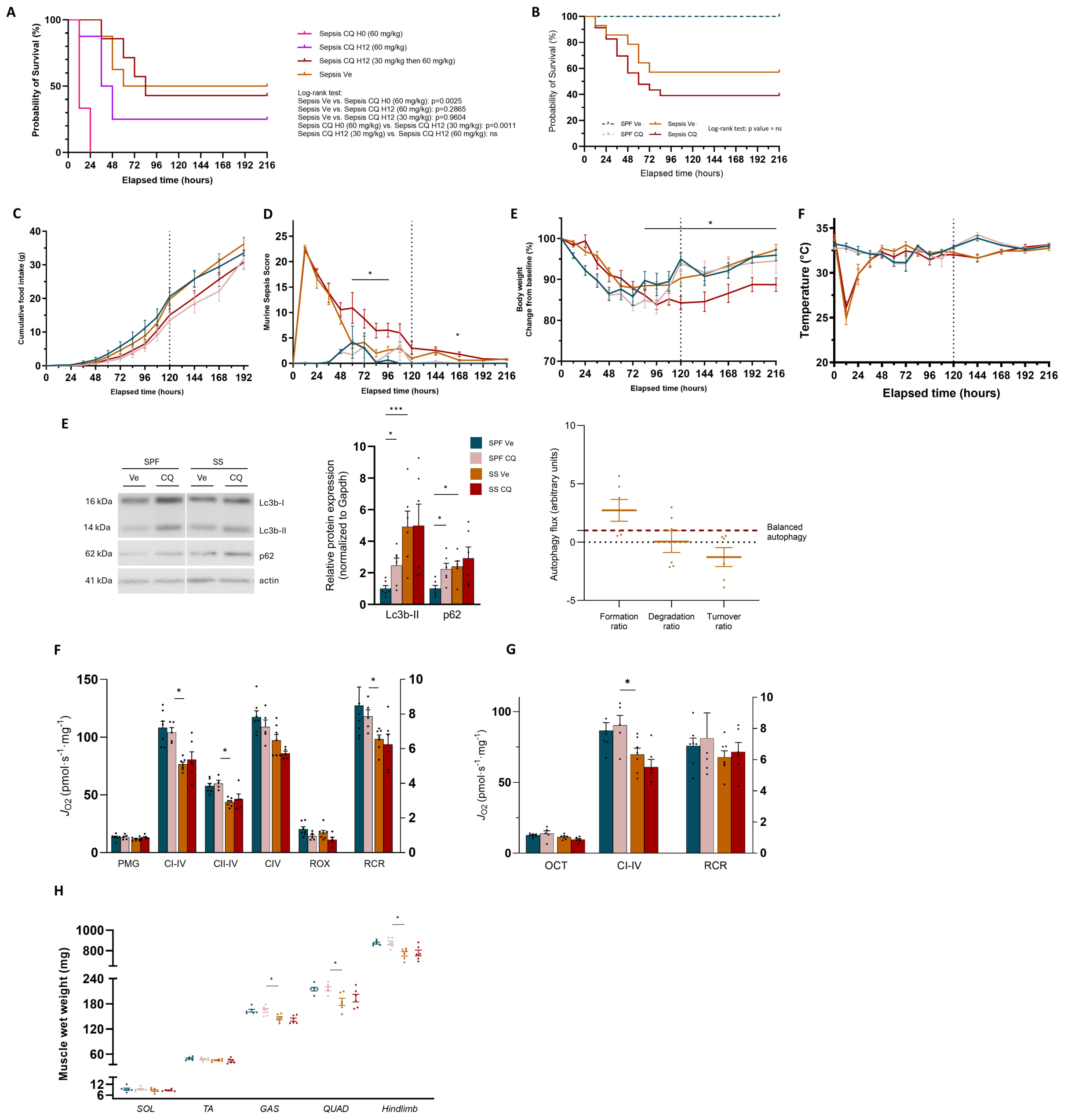

Figure S5. Murine CSI-induced sepsis model with autophagy blockade. A, Kaplan-Meier survival curve for different doses and times of CQ administration in SS mice: H0 pre-treatment (n = 3), H12 delayed treatment with first dose of 60 mg/kg (n = 8), H12 delayed treatment with first dose of 30 mg/kg (n = 7), without CQ (n = 8). Summary of the

log-rank test results (right). **B,** Kaplan-Meier survival curve for SPF Ve mice (n = 6), SPF CQ mice (n = 7), Sepsis Ve mice (n = 14) and Sepsis CQ mice (n = 23). **C-F,** Cumulative food intake (**C**), Murine Sepsis Score **(D**), change in body weight (**E**), and temperature (**F**) for SPF Ve mice (n = 6), SPF CQ mice (n = 7), SS Ve mice (n = 8), SS CQ mice (n = 9). **G**, Protein expression of Lc3b-II and P62 in quadriceps (n = 6 for each group) and calculation of the autophagy flux based on Lc3b-II expression. **H-I**, PMG- (**H**) and Oct-M- (**I**) linked J_{O2} of *Soleus* pmf, as previously described (n = 5 – 7 for each group). One missing data for PMG-linked CII-IV for SPF CQ mice due to a technical issue. **J,** Muscle wet weight: *SOL*, *TA*, *GAS*, and *QUAD* refer to *Soleus*, *Extensor Digitorum Longus*, *Tibialis Anterior*, *Gastrocnemius*, and quadriceps, respectively, and the term “hindlimb” refers to the sum of the *SOL*, *TA*, *GAS*, and *QUAD* of both limbs. SPF, sham mice fed *ad libitum*; SS, sepsis-surviving mice; Ve, vehicle injection; CQ, chloroquine injection. SPF Ve in dark blue, SPF CQ in light red, SS Ve in orange, SS CQ in dark red. Each symbol represents one animal. Data expressed as mean values with SEM. Data analyzed by log-rank test (**A-B**), two-way ANOVA test (**C-F**) and Kruskal-Wallis test with post-hoc Dunn’s test (**E-H**).

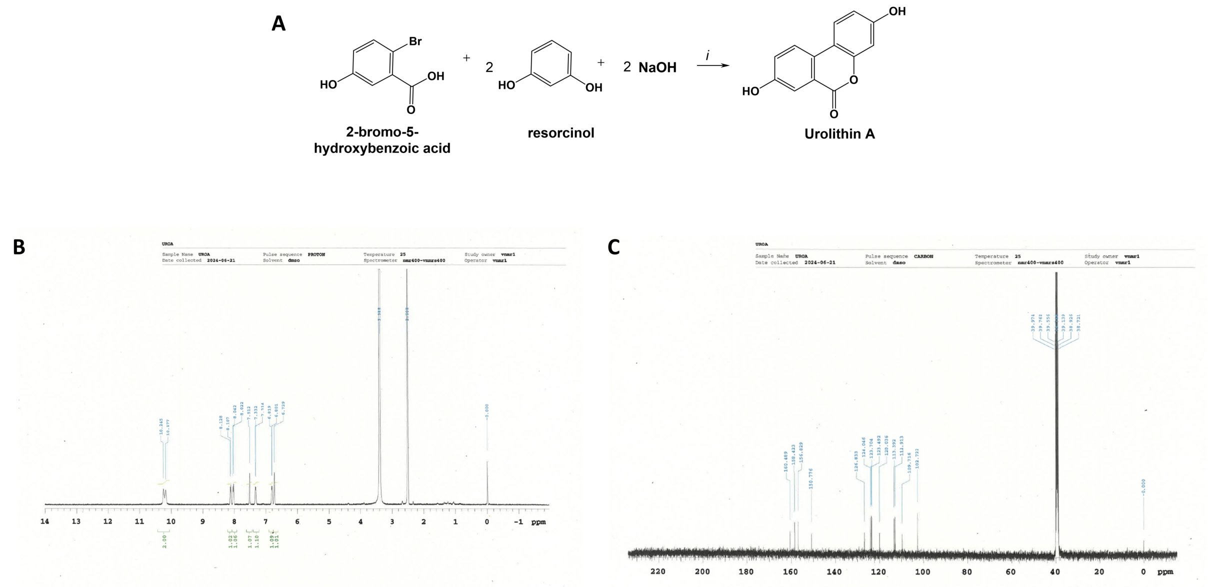

Figure S6. Green synthesis of Urolithin A. A, Green Hurtley reaction: 2-bromo-5-hydroxybenzoic acid was stirred in aqueous sodium hydroxide with resorcinol for 30 min at 50 °C; the resulting mixture was then heated to 100 °C for 3 hours in presence of catalytic amount of Ecocat-MG2 obtained from *Miscanthus x giganteus* or $CuSO_4$. **B-C**, ^{1}H (**B**) and ^{13}C (**C**) NMR spectrum of urolithin A. Nuclear magnetic resonance (NMR) spectra were acquired at room temperature (RT) at 400 MHz for ^{1}H NMR, and at 100 MHz for ^{13}C NMR, on a Varian 400-MR spectrometer with tetramethylsilane (TMS) as internal standard. Chemical shifts (δ) are expressed in ppm relative to TMS. Splitting patterns are designated: s, singlet; d, doublet; br s, broaden singlet. Coupling constants (*J*) are reported in Hertz (Hz).

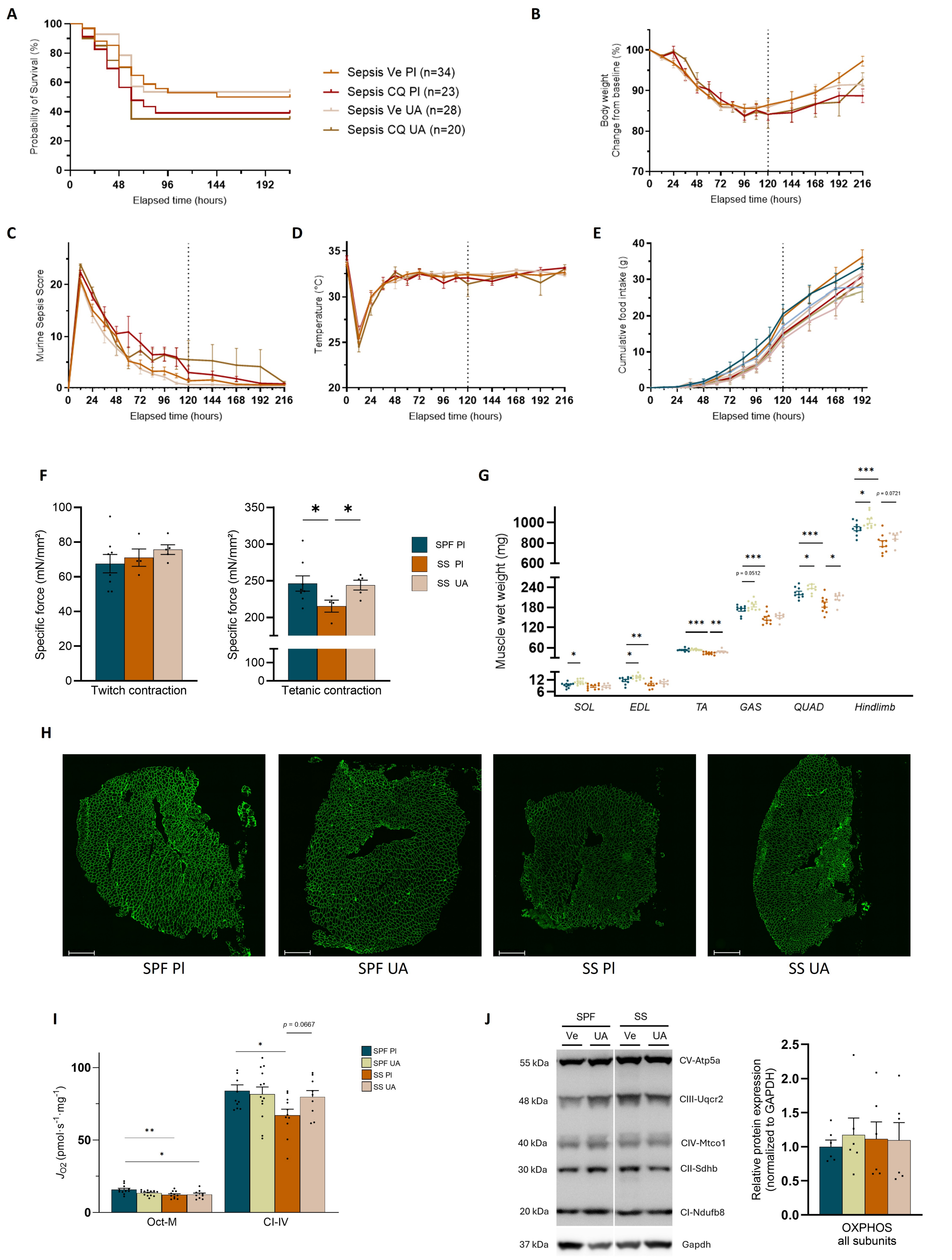

Figure S7. Murine CSI-induced sepsis model with urolithin A: clinical characteristics and muscle phenotype. A, Kaplan-Meier survival curve of Sepsis Pl (n = 34), Sepsis CQ (n = 23), Sepsis UA (n = 28) and Sepsis CQ UA (n = 20) mice. **B-E**, Changes in body weight (**B**), Murine Sepsis Score (**C**), temperature (**D**) and cumulative food intake (**E**) for SS Ve Pl (n = 18), SS CQ Pl (n = 14), SS Ve UA (n = 14), SS CQ UA (n = 7) mice. **F**, *Ex vivo* contractility

of Soleus: specific twitch force (left), specific tetanic force (right) (n = 4 – 8 for each group). **G**, Muscle wet weight for SPF Pl mice (n = 10), SPF UA mice (n = 10), SS Pl mice (n = 10), SS UA mice (n = 8). *SOL*, *TA*, *GAS*, and *QUAD* refer to *Soleus*, *Extensor Digitorum Longus*, *Tibialis Anterior*, *Gastrocnemius*, and quadriceps, respectively, and the term "hindlimb" refers to the sum of the *SOL*, *TA*, *GAS*, and *QUAD* of both limbs. **H**. Representative images of *Tibialis anterior* laminin immunofluorescence staining observed with Axioscan, SPF Pl (left), SPF UA (middle left), SS Pl (middle right), SS UA (right). Scale bars, 100 µm. **I**, Oct-M linked J_{O2} in *Soleus* pmf for SPF Pl (n = 11), SPF UA (n = 13), SS Pl (n = 11), SS UA (n = 10). **J**, Protein expression of 5 respiratory chain subunits (n = 6 for each group). SPF, sham pair-fed mice; SS, sepsis-surviving mice; Ve, vehicle; Pl, placebo; CQ, chloroquine; UA, urolithin A. SPF Pl in dark blue, SPF UA in light yellow, SPF CQ in light red, SPF CQ UA in dark yellow, SS Pl in orange, SS UA in light orange, SS CQ UA in dark brown. Each symbol represents one animal. Data expressed as mean values with SEM. Data analyzed by log rank test (**A**), two-way ANOVA test (**B-E**), one-way ANOVA test with post-hoc Fisher's LSD test (**G**, **I**), and Kruskal-Wallis test with post-hoc Dunn's test (**F**, **J**).

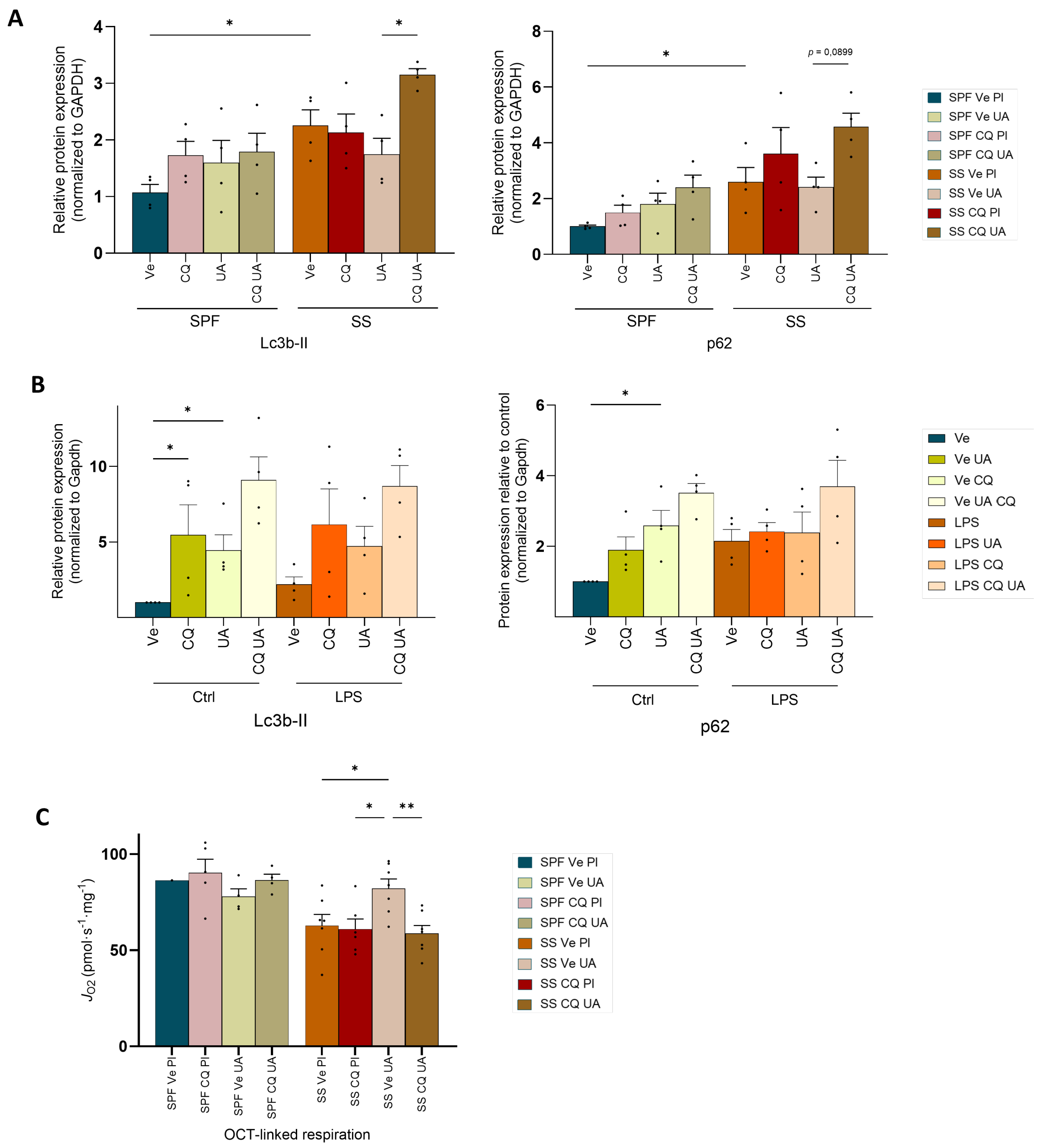

Figure S8. Murine CSI-induced sepsis model with urolithin A: autophagy flux. A, Protein expression of Lc3b-II (left) and p62 (right) according to UA treatment with or without autophagy blockade by CQ in SPF and SS mice (n = 4 for each group). **B**, Protein expression of Lc3b-II (left) and p62 (right) according to UA and CQ incubation in differentiated C2C12 cells challenged by LPS (n = 4 for each group). **C,** Oct-M linked J_{O2} in *Soleus* pmf for SPF Ve Pl (n = 1), SPF Ve UA (n = 4), SPF CQ Pl (n = 5), SPF CQ UA (n = 4), SS Ve Pl (n = 7), SS Ve UA (n = 7), SS CQ Pl (n = 6), SS CQ UA (n = 7). SPF, sham pair-fed mice; SS, sepsis-surviving mice; Ve, vehicle; Pl, placebo; CQ, chloroquine; UA, urolithin A. SPF Pl in dark blue, SPF UA in light yellow, SPF CQ in light red, SPF CQ UA in dark yellow, SS Pl in orange, SS UA in light orange, SS CQ UA in dark brown. Each symbol represents one

animal. Data expressed as mean values with SEM. Data analyzed with Kruskal-Wallis test with post-hoc Dunn's test.

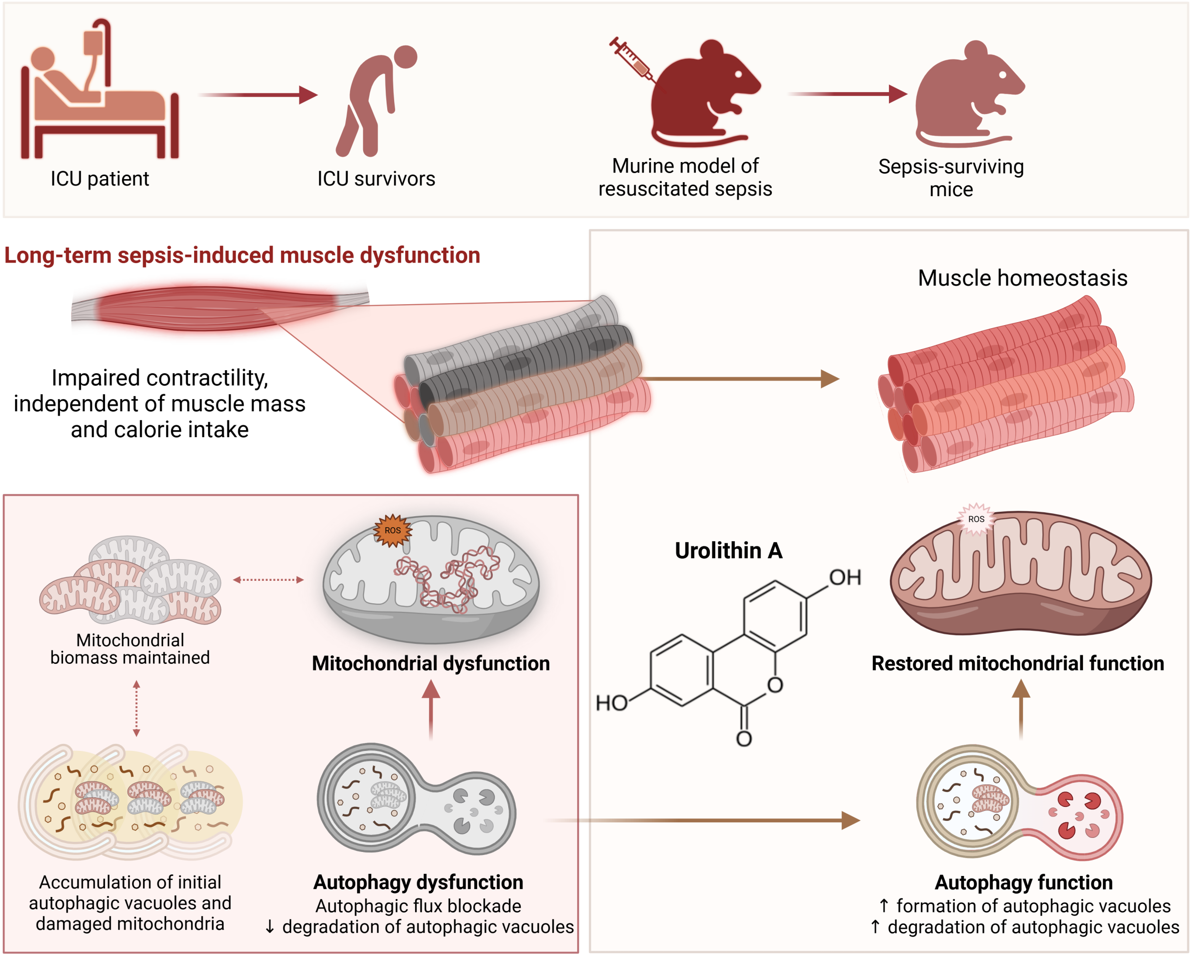

Figure S9. Visual abstract.

Supplementary Tables

Table S1. Description of the Murine Sepsis Score. According to Shrum *et al.* BMC Res Notes 2014 (DOI: 10.1186/1756-0500-7-233).

Variable	Score and description
Appearance	0- Coat is smooth.
	1- Patches of hair piloerected.
	2- Majority of back is piloerected.
	3- Piloerection may or may not be present, mouse appears "puffy".
	4- Piloerection may or may not be present, mouse appears emaciated.
Level of consciousness	0- Mouse is active.
	1- Mouse is active but avoids standing upright.
	2- Mouse activity is noticeably slowed. The mouse is still ambulant.
	3- Activity is impaired. Mouse only moves when provoked, movements have a tremor.
	4- Activity severely impaired. Mouse remains stationary when provoked, with possible tremor.
Activity	0- Normal amount of activity. Mouse is any of: eating, drinking, climbing, running, fighting.
	1- Slightly suppressed activity. Mouse is moving around bottom of cage.
	2- Suppressed activity. Mouse is stationary with occasional investigative movements.
	3- No activity. Mouse is stationary.
	4- No activity. Mouse experiencing tremors, particularly in the hind legs.
Response to stimulus	0- Mouse responds immediately to auditory stimulus or touch.
	1- Slow or no response to auditory stimulus; strong response to touch (moves to escape).
	2- No response to auditory stimulus; moderate response to touch (moves a few steps).
	3- No response to auditory stimulus; mild response to touch (no locomotion).

	4- No response to auditory stimulus. Little or no response to touch. Cannot right itself if pushed over.	
Eyes	0- Open.	
	1- Eyes not fully open, possibly with secretions.	
	2- Eyes at least half closed, possibly with secretions.	
	3- Eyes half closed or more, possibly with secretions.	
	4- Eyes closed or milky.	
Respiration rate	0- Normal, rapid mouse respiration.	
	1- Slightly decreased respiration (rate not quantifiable by eye).	
	2- Moderately reduced respiration (rate at the upper range of quantifying by eye).	
	3- Severely reduced respiration (rate easily countable by eye, 0.5 s between breaths).	
	4- Extremely reduced respiration (>1 s between breaths).	
Respiration quality	0- Normal.	
	1- Brief periods of laboured breathing.	
	2- Laboured, no gasping.	
	3- Laboured with intermittent gasps.	
	4- Gasping.	

Table S2. Description of the antibodies used in Western-blot and Immunofluorescence experiments.

Antibodies	Reference	Dilution
GAPDH	#2118S Cell Signaling	1/5000
OXPHOS rodent cocktail	Ab110413 Abcam	1/1000
PINK1	BC100-494 Novus	1/1000
PARKIN	Ab77924 Abcam	1/1000
P62	#5114 Cell Signaling	1/1000
LC3B	Ab192890 Abcam	1/2000
LAMININ-A2	Sc-59854 Santa Cruz	1/100
MYHC I	BA-D5 DSHB	1/50
MYHC IIA	SC-71 DSHB	1/50
MYHC IIB	BF-F3 DSHB	1/50
8-OXOGUANINE	Ab206461 Abcam	1/50
VDAC1/3	Ab14734 Abcam	1/100
3-NITROTYROSINE	Ab61382 Abcam	1/1000
NDUFB8	Ab192878 Abcam	1/500
Mouse IgG	#7076S Cell Signaling	1/4000
Rabbit IgG	#7074S Cell Signaling	1/4000
Mouse IgG1 AlexaFluor 488	A-21121 Invitrogen	1/250
Mouse IgM AlexaFluor 555	A-21426 Invitrogen	1/250
Mouse IgG2b AlexaFluor 647	A-21242 Invitrogen	1/250
Rat IgG AlexaFluor 555	A-21434 Invitrogen	1/250
Rat IgG AlexFluor 405	A-48261 Invitrogen	1/250

Table S3. Description of the primers used in RTqPCR experiments (relative RNA expression).

Gene	Sequence: forward	Sequence: reverse	NCBI reference sequence
Ppia	GCGTCTCCTTCGAGCTGTTT	GCGTGTAAAGTCACCACCCT	NM_008907.2
Lc3b	CACTGCTCTGTCTTGTGTAGGTTG	TCGTTGTGCCTTTATTAGTGCATC	NM_026160.5
P62	GCTGAAGGAAGCTGCCCTAT	TTGGTCTGTAGGAGCCTGGT	NM_011018.3
Pink	TTGCAATGCCGCTGTGTATG	TGGAGGAACCTGCCGAGATA	NM_026880.2
Parkin	CCCGGTGACCATGATAGTGTT	TGAACCGTCAGGTGATTCGG	NM_016694.4

Table S4. Description of the primers used in qPCR experiments (mitochondrial DNA copy number).

Gene	Sequence: forward	Sequence: reverse	NCBI reference sequence
Ppia	ACACGCCATAATGGCACTGG	CAGTCTTGGCAGTGCAGAT	NM_008907.2
Nd1	TCCGAGCATCTTATCCACGC	GTATGGTGGTACTCCCGCTG	NC_005089.1
Nd2	ATCCTCCTGGCCATCGTACT	ATCAGAAGTGGAATGGGGCG	NC_005089.1

Uncropped western blots

The red boxes indicate the cropped images shown in the main figure.

Figure 3B

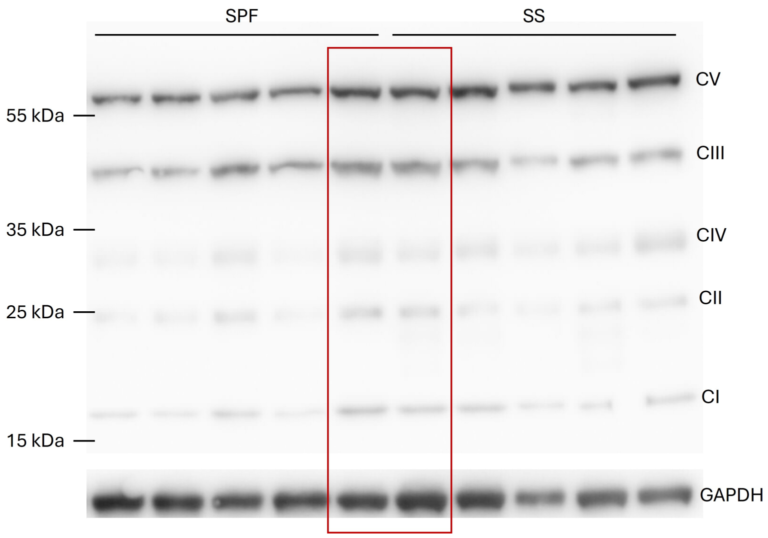

Figure 4C

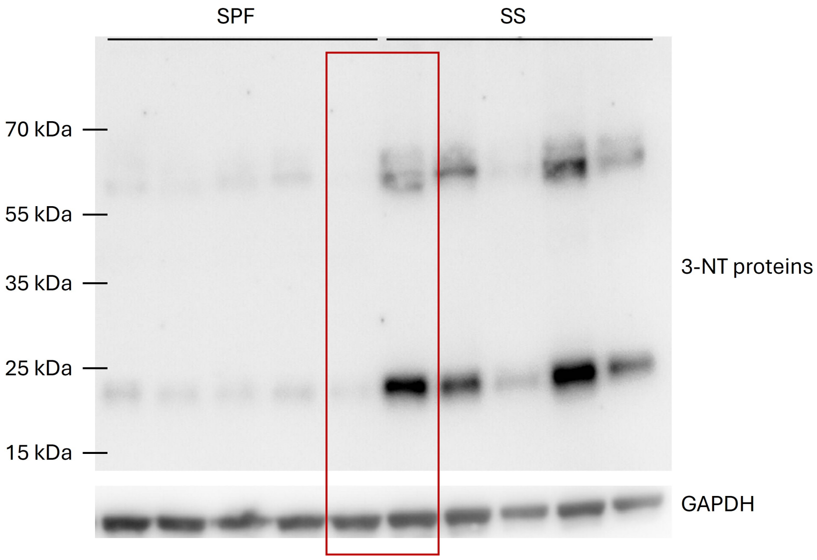

Figure 4D

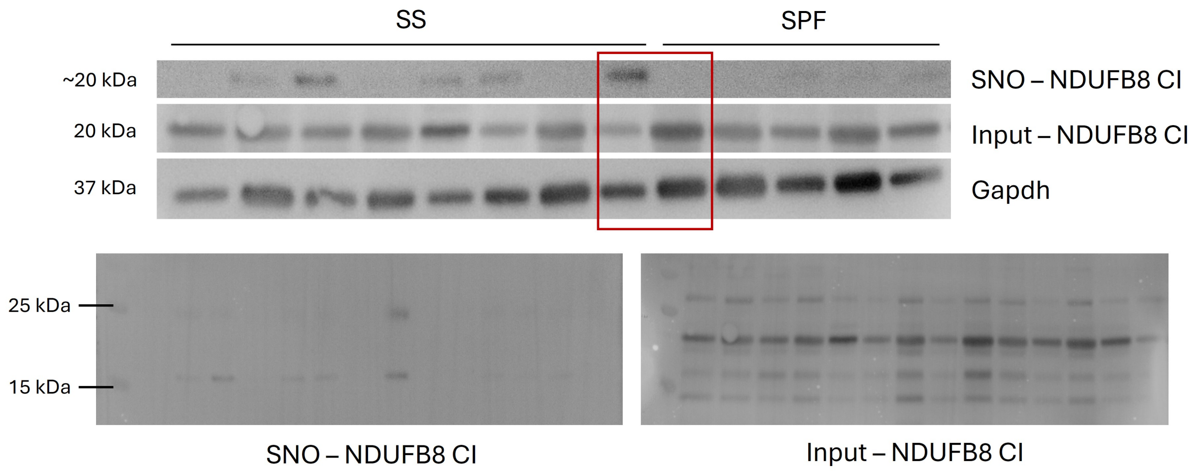

The images were rotated 180 degrees in the main figure to maintain consistency in the presentation of the blots (SPF on the left and SS on the right); however, the samples were originally loaded into the left wells for SPF and the right wells for SS, as shown in the original image.

Figure 4E (left)

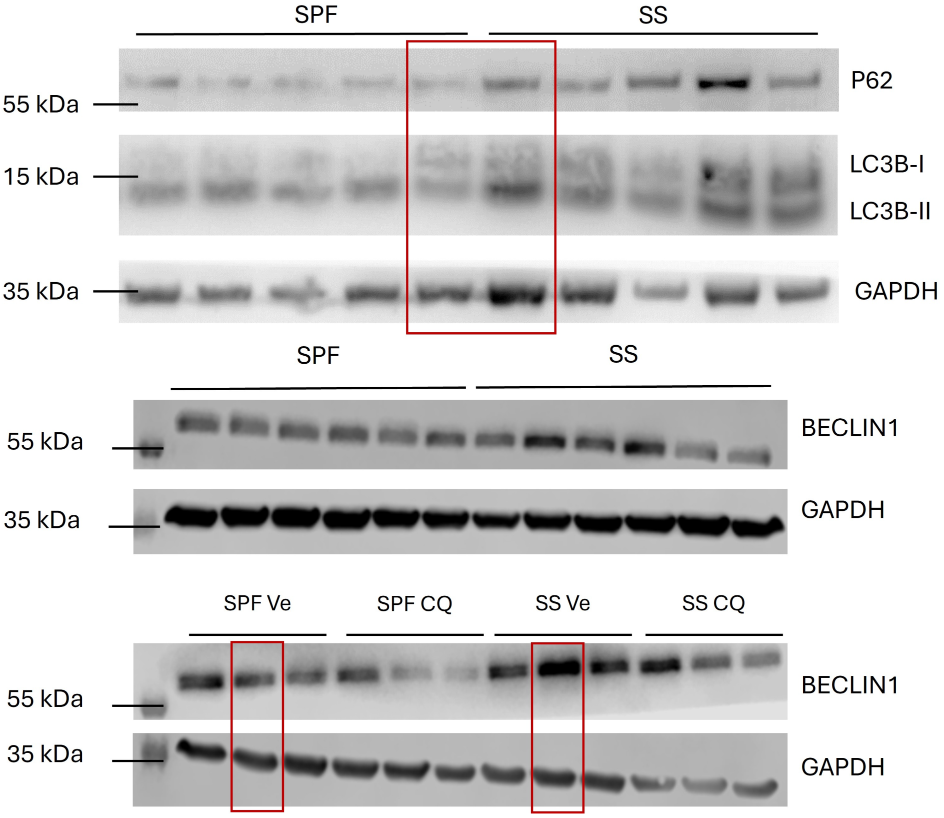

Figure 4E (right)

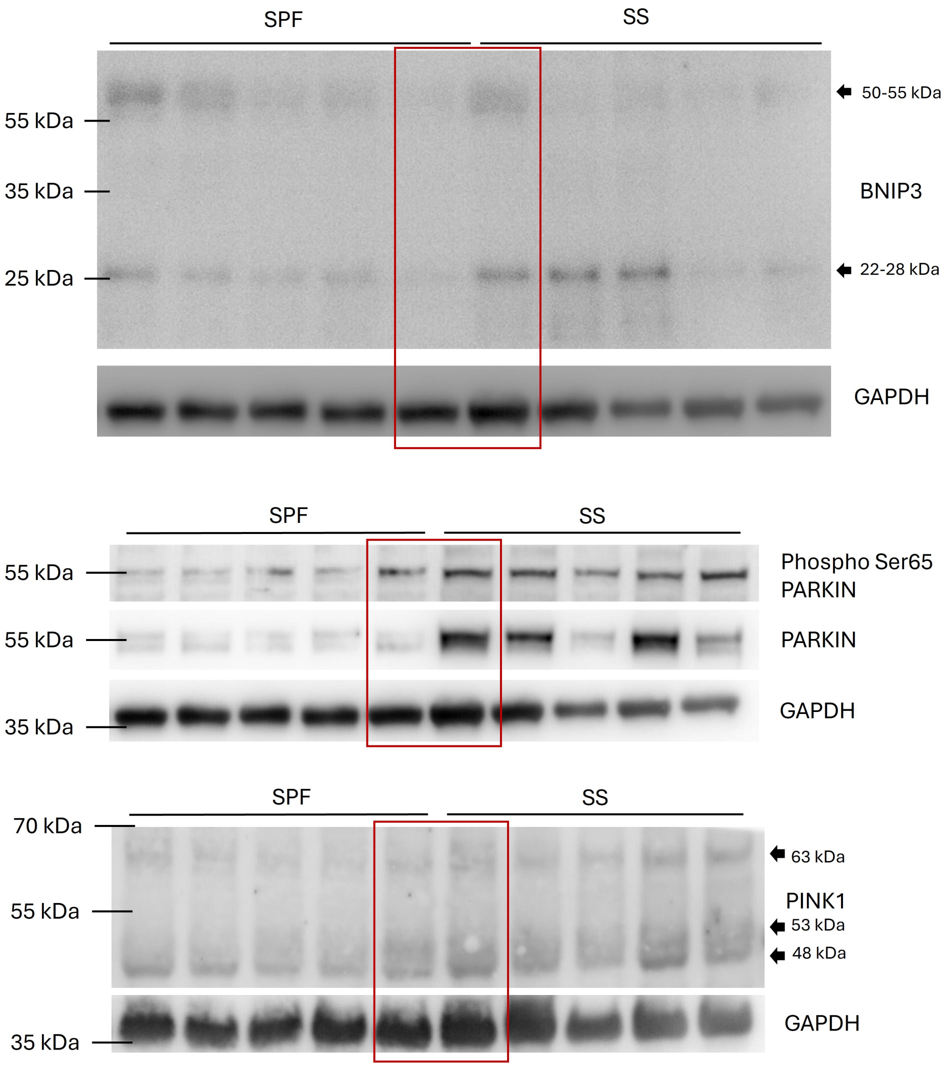

Figure 5D

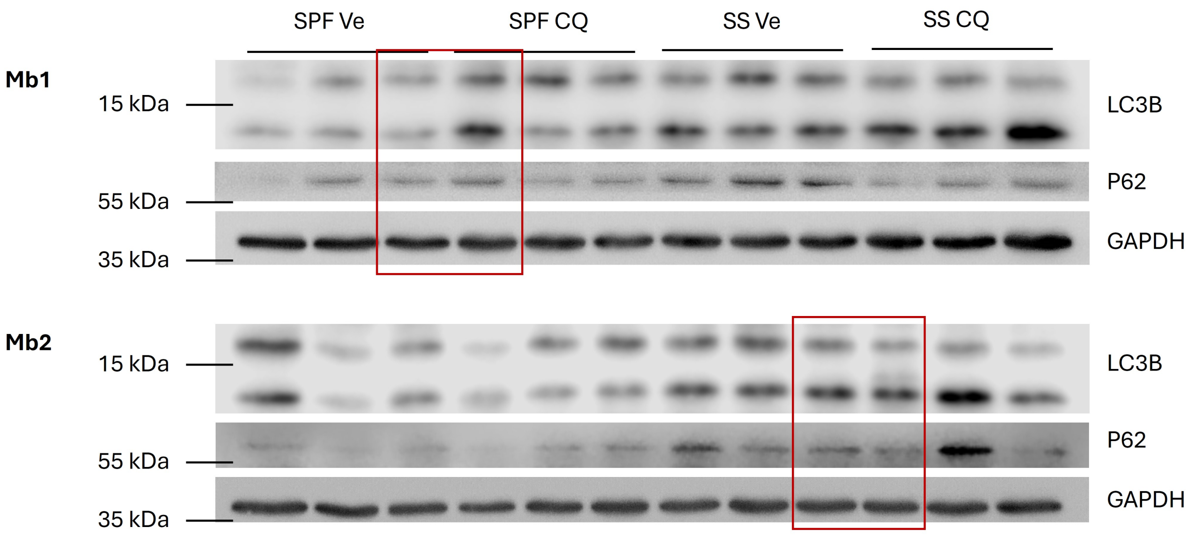

Figure 5F

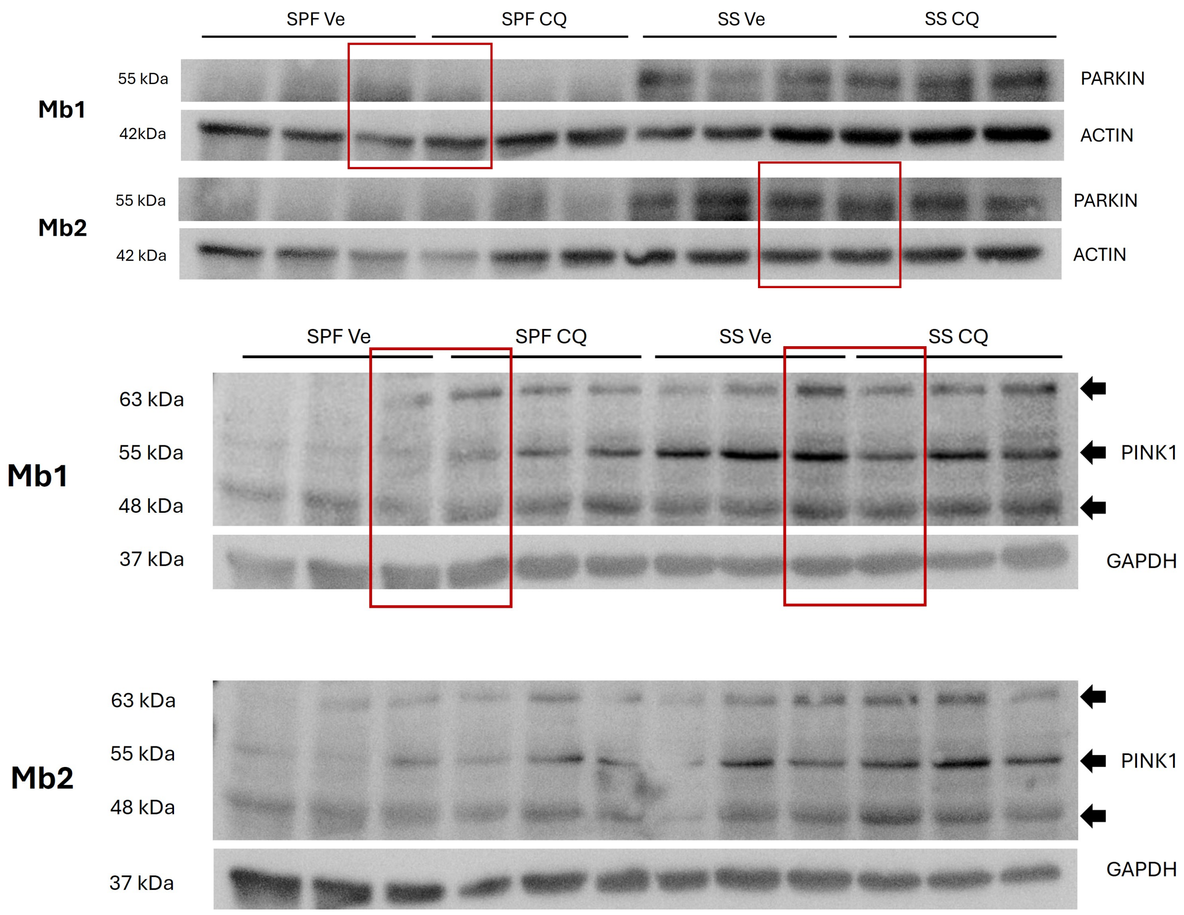

Figure S5E

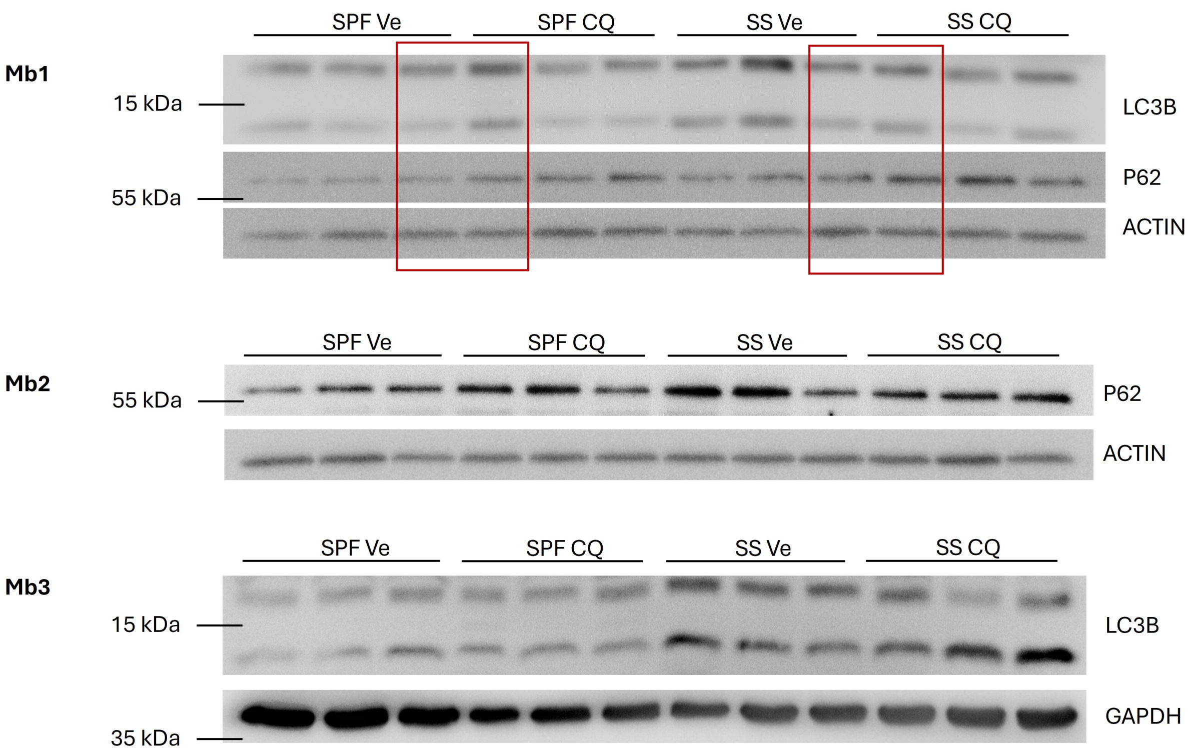

Figure 6E

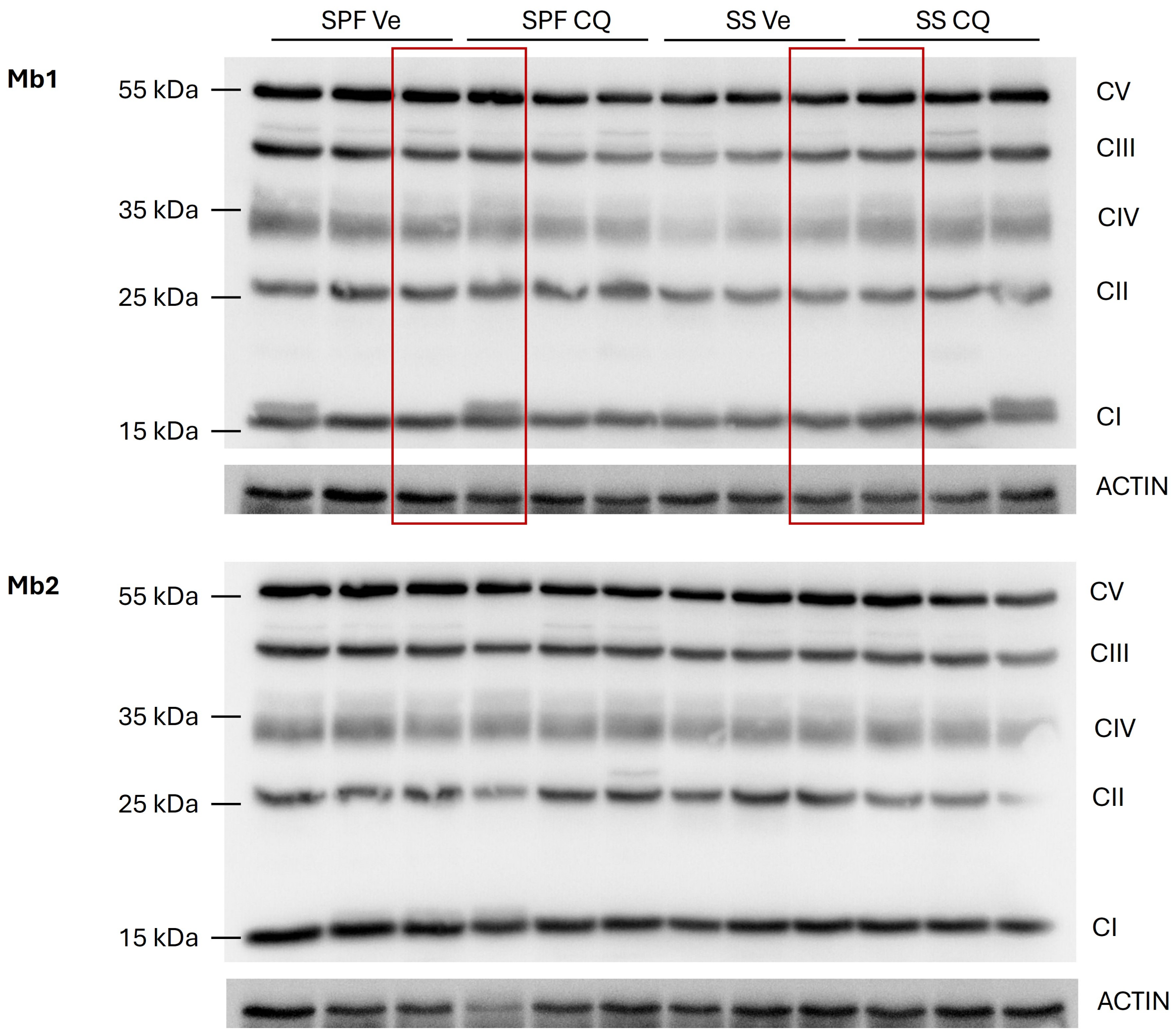

Figure 7E

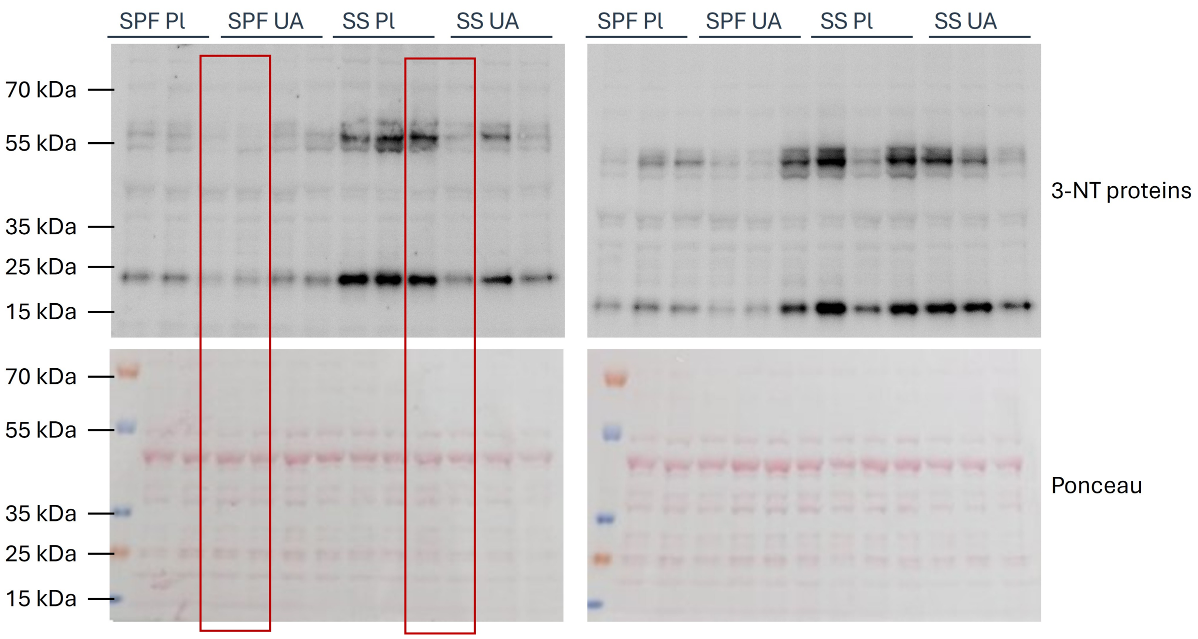

Figure S7H

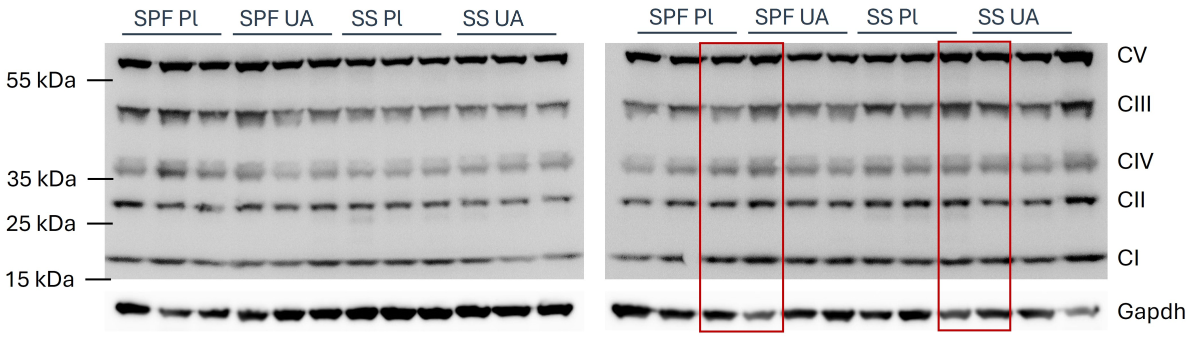

SPF PL
SPF UA
SS PL
SS UA
SPF PL
SPF UA
SS PL
SS UA
55 kDa
35 kDa
25 kDa
15 kDa
CV
CIII
CIV
CII
CI
Gapdh

Figure 8A

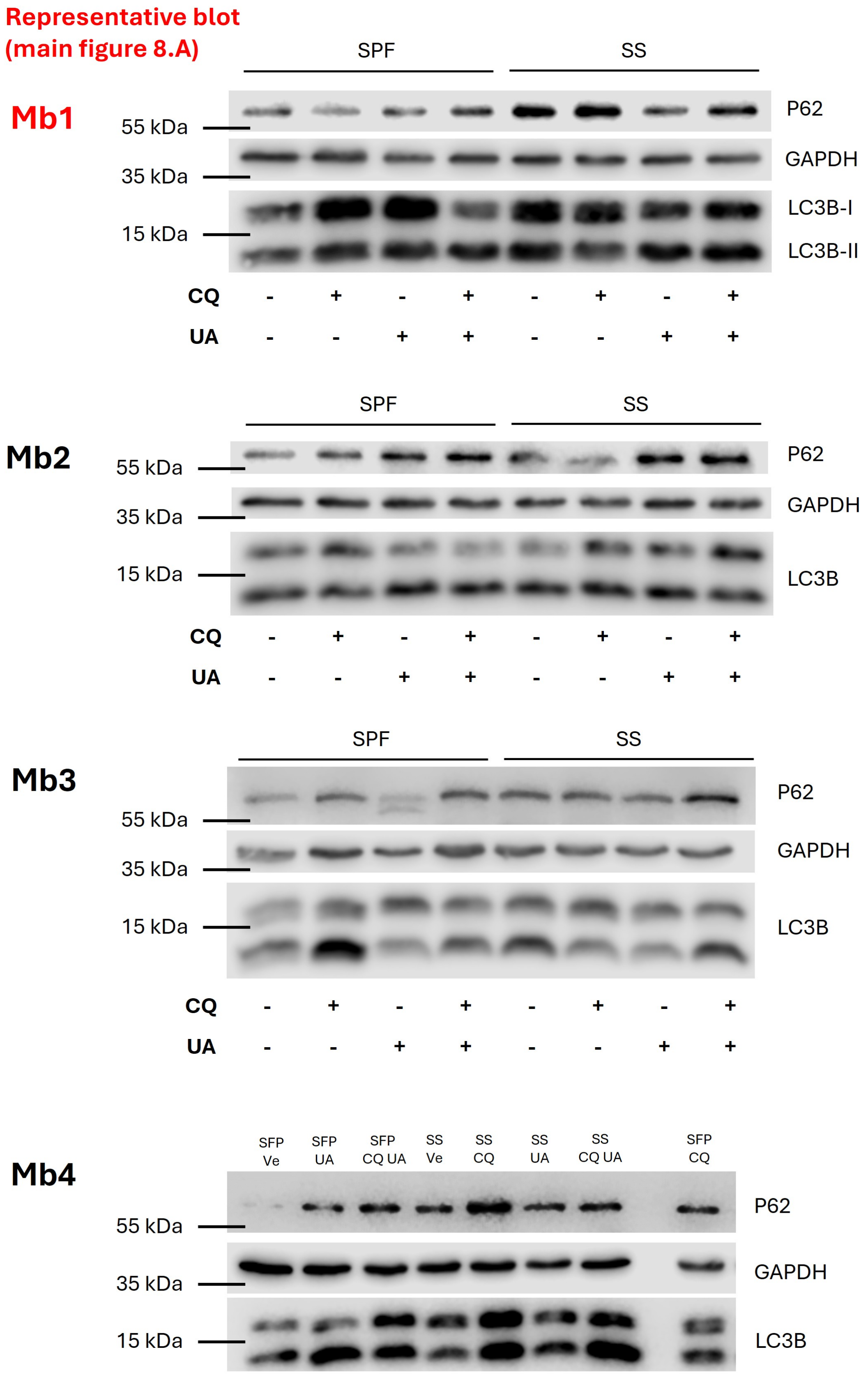
Representative blot
(main figure 8.A)
Mb1
SPF
SS
55 kDa
35 kDa
15 kDa
P62
GAPDH
LC3B-I
LC3B-II
CQ - + - + - + - +
UA - - + + - - + +
Mb2
SPF
SS
55 kDa
35 kDa
15 kDa
P62
GAPDH
LC3B
CQ - + - + - + - +
UA - - + + - - + +
Mb3
SPF
SS
55 kDa
35 kDa
15 kDa
P62
GAPDH
LC3B
CQ - + - + - + - +
UA - - + + - - + +
Mb4
SFP Ve
SFP UA
SFP CQ UA
SS Ve
SS CQ
SS UA
SS CQ UA
SFP CQ
55 kDa
35 kDa
15 kDa
P62
GAPDH
LC3B

Figure 8B

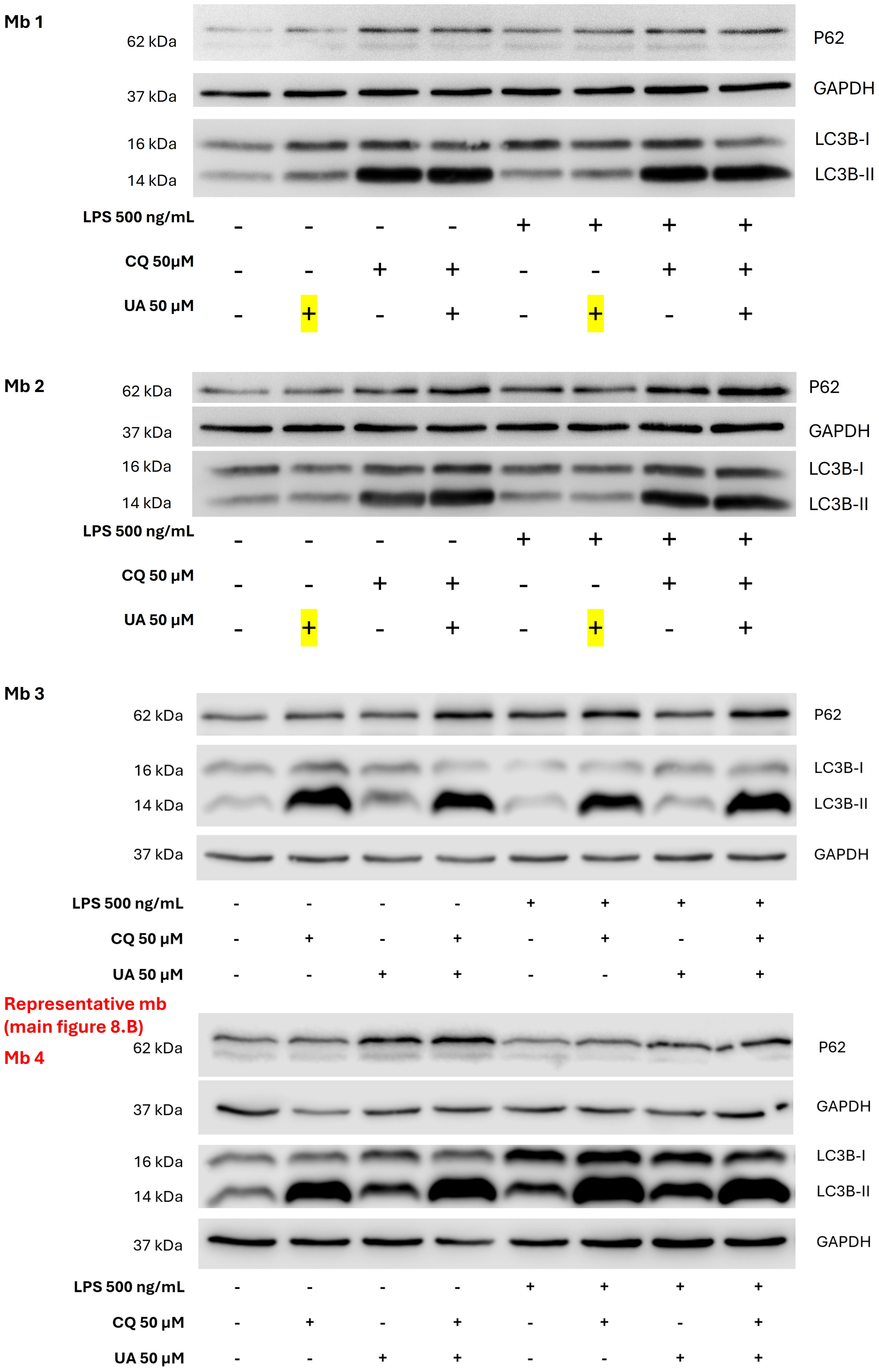
Mb 1
62 kDa
37 kDa
16 kDa
14 kDa
P62
GAPDH
LC3B-I
LC3B-II
LPS 500 ng/mL - - - - + + + +
CQ 50µM - - + + - - + +
UA 50 µM - + - + - + - +
Mb 2
62 kDa
37 kDa
16 kDa
14 kDa
P62
GAPDH
LC3B-I
LC3B-II
LPS 500 ng/mL - - - - + + + +
CQ 50 µM - - + + - - + +
UA 50 µM - + - + - + - +
Mb 3
62 kDa
16 kDa
14 kDa
37 kDa
P62
LC3B-I
LC3B-II
GAPDH
LPS 500 ng/mL - - - - + + + +
CQ 50 µM - + - + - + - +
UA 50 µM - - + + - - + +
Representative mb
(main figure 8.B)
Mb 4
62 kDa
37 kDa
16 kDa
14 kDa
37 kDa
P62
GAPDH
LC3B-I
LC3B-II
GAPDH
LPS 500 ng/mL - - - - + + + +
CQ 50 µM - + - + - + - +
UA 50 µM - - + + - - + +

The loading order of the UA and CQ conditions was changed on membranes 1 and 2 compared to membranes 3 and 4. To draw the reader's attention to this point, the UA conditions on membranes 1 and 2 (which differ from those on membranes 3 and 4) have been highlighted in yellow.

Supplementary references

1. Walsh CJ, Batt J, Herridge MS, Mathur S, Bader GD, Hu P, et al. Transcriptomic analysis reveals abnormal muscle repair and remodeling in survivors of critical illness with sustained weakness. Sci Rep. 2016 Jul 14;6:29334.

2. Rath S, Sharma R, Gupta R, Ast T, Chan C, Durham TJ, et al. MitoCarta3.0: an updated mitochondrial proteome now with sub-organelle localization and pathway annotations. Nucleic Acids Research. 2021 Jan 8;49(D1):D1541–7.

3. Starr ME, Steele AM, Saito M, Hacker BJ, Evers BM, Saito H. A new cecal slurry preparation protocol with improved long-term reproducibility for animal models of sepsis. PLoS One. 2014;9(12):e115705.

4. Steele AM, Starr ME, Saito H. Late Therapeutic Intervention with Antibiotics and Fluid Resuscitation Allows for a Prolonged Disease Course with High Survival in a Severe Murine Model of Sepsis. Shock. 2017;47(6):726–34.

5. Pierre A, Bourel C, Favory R, Brassart B, Wallet F, Daussin FN, et al. Sepsis-like Energy Deficit Is Not Sufficient to Induce Early Muscle Fiber Atrophy and Mitochondrial Dysfunction in a Murine Sepsis Model. Biology. 2023 Apr;12(4):529.

6. Mauthe M, Orhon I, Rocchi C, Zhou X, Luhr M, Hijlkema KJ, et al. Chloroquine inhibits autophagic flux by decreasing autophagosome-lysosome fusion. Autophagy. 2018 Jul 20;14(8):1435–55.

7. Eenige R van, Verhave PS, Koemans PJ, Tiebosch IACW, Rensen PCN, Kooijman S. RandoMice, a novel, user-friendly randomization tool in animal research. PLOS ONE. 2020 Aug 5;15(8):e0237096.

8. Guérin T, Waterlot C, Lipka E, Gervois P, Bulteel D, Betrancourt D, et al. Ecocatalysed Hurtley reaction: Synthesis of urolithin derivatives as new potential RAGE antagonists with anti-ageing properties. Sustainable Chemistry and Pharmacy. 2021 Oct;23:100518.

9. Pesta D, Gnaiger E. High-resolution respirometry: OXPHOS protocols for human cells and permeabilized fibers from small biopsies of human muscle. Methods Mol Biol. 2012;810:25–58.

10. Doerrier C, Garcia-Souza LF, Krumschnabel G, Wohlfarter Y, Mészáros AT, Gnaiger E. High-Resolution FluoRespirometry and OXPHOS Protocols for Human Cells, Permeabilized Fibers from Small Biopsies of Muscle, and Isolated Mitochondria. Methods Mol Biol. 2018;1782:31–70.

11. Boulinguiez A, Duhem C, Mayeuf-Louchart A, Pourcet B, Sebti Y, Kondratska K, et al. NR1D1 controls skeletal muscle calcium homeostasis through myoregulin repression. JCI Insight. 2022 Sep 8;7(17):e153584.

12. Plaza-Zabala A, Sierra-Torre V, Sierra A. Assessing Autophagy in Microglia: A Two-Step Model to Determine Autophagosome Formation, Degradation, and Net Turnover. Front Immunol [Internet]. 2021 Jan 29 [cited 2024 Jul 8];11. Available from: https://www.frontiersin.org/journals/immunology/articles/10.3389/fimmu.2020.620602/full

13. Jaffrey SR, Snyder SH. The Biotin Switch Method for the Detection of S-Nitrosylated Proteins. Science's STKE. 2001 Jun 12;2001(86):pl1–pl1.

14. Forrester MT, Foster MW, Benhar M, Stamler JS. Detection of Protein S-Nitrosylation with the Biotin Switch Technique. Free Radic Biol Med. 2009 Jan 15;46(2):119–26.

15. Danckaert A, Trignol A, Le Loher G, Loubens S, Staels B, Duez H, et al. MuscleJ2: a rebuilding of MuscleJ with new features for high-content analysis of skeletal muscle immunofluorescence slides. Skelet Muscle. 2023 Aug 23;13(1):14.

16. Ohno M, Oka S, Nakabeppu Y. Quantitative analysis of oxidized guanine, 8-oxoguanine, in mitochondrial DNA by immunofluorescence method. Methods Mol Biol. 2009;554:199–212.
